# The Azide-Allene Dipolar Cycloaddition: Is DFT Able to Predict Site- and Regio-Selectivity?

**DOI:** 10.3390/molecules26040928

**Published:** 2021-02-10

**Authors:** Giorgio Molteni, Alessandro Ponti

**Affiliations:** 1 Dipartimento di Chimica, Università degli Studi di Milano, via Golgi 19, 20133 Milano, Italy; giorgio.molteni@unimi.it; 2 Istituto di Scienze e Tecnologie Chimiche “Giulio Natta” (SCITEC), Consiglio Nazionale delle Ricerche, via Golgi 19, 20133 Milano, Italy

**Keywords:** 1,3-dipolar cycloaddition, allene, azide, M08-HX, local softness

## Abstract

The site- and regio-selectivity of thermal, uncatalysed 1,3-dipolar cycloadditions between arylazides and mono- or tetra-substituted allenes with different electronic features have been investigated by both conceptual (reactivity indices) and computational (M08-HX, ωB97X-D, and B3LYP) DFT approaches. Both approaches show that these cycloadditions follow a nonpolar one-step mechanism. The experimental site- and regio-selectivity of arylazides towards methoxycarbonyl- and sulfonyl-allenes as well as tetramethyl- and tetrafluoro-allenes was calculated by DFT transition state calculations, achieving semiquantitative agreement to both previous and novel experimental findings. From the mechanistic standpoint, ^1^H-NMR evidence of a methylene-1,2,3-triazoline intermediate reinforces the reliability of the computational scheme.

## 1. Introduction

Organic azides have experienced a renaissance since 2001, when Sharpless [1] and Maldal [2] independently disclosed the way to the regioselective azide-alkyne cycloaddition based upon the “click” approach [3,4]. A number of mechanistic and theoretical studies on this topic have been developed since then [5,6]. Curiously enough, the theoretical interpretation of thermal processes involving the site- and regio-selectivity of the azide cycloaddition [7,8] was the object of a lesser effort [9]. It is fair to say that, for the majority of organic chemists, the prediction of the behaviour of azides towards double-bonded dipolarophiles relies upon the simple FMO (Frontier Molecular Orbital) [10] framework outstandingly depicted by Houk in the early seventies [11,12]. This statement is also true in the case of cumulated carbon-carbon double bonds [13], but is should be kept in mind that arylazide cycloaddition can often be controlled by both the HOMO and the LUMO of the 1,3-dipole, and the observed regioselectivities are difficult to rationalize on the grounds of the FMO theory. As a further difficulty, both allene double bonds are suitable sites for dipolar attack, and a poor rationale of the cycloaddition sites-electivity (preference for one of the double bonds) and regioselectivity (preference for one of the relative reactants’ orientation) was given in the case of both nitrilium [14,15] and azonium [16] betaines, including organic azides.

The computational chemistry of allenes has been reviewed up to 2013 [17]. A few studies investigated the regioselectivity of 1,3-dipolar cycloadditions of allenes by calculating the activation barriers of the pathways leading to the isomeric products (these reactions usually are under kinetic control). In the first such study, MP4SDTQ/6-31G* calculation of the diazomethane + allene (1,2-propadiene) cycloaddition transition structures (TSs) supported the one-step mechanism, and the calculated barriers indicated the major isomer in agreement with experiment [18]. The study was extended to fluoro- and 1,1-difluoroallene. MP4SDTQ/6-31G* calculations reproduced the experimental order of the isomer yields. Results were less satisfactory when using formonitrileoxide as a dipole [19]. DFT calculations at the B3LYP/6-31G(d) level of the cycloaddition of allene to diazomethane, formonitrile oxide, and methylene nitrone supported a stepwise mechanism and indicated the major isomer of the diazomethane + allene cycloaddition in agreement with experiment [20]. B3LYP/6-31G(d,p) calculations of the reactions between *N*-heterocyclic carbene-derived C-C-S 1,3-dipoles and 4-benzyl-1-methoxycarbonylallene indicated the major isomer in agreement with experiment [21]. B3LYP/6-31G(d,p) calculations of the cycloaddition between arylnitrileoxides and arylallenes reproduced the site- and regioselectivity of these reactions with semiquantitative accuracy (less than 10% error in both cases) [22].

A complementary computational approach to reactivity uses indices defined in the framework of conceptual DFT [23,24]. Global reactivity indices, such as the electron chemical potential μ and hardness η [25], softness *S* [26], and the electrophilicity ω [27,28] and nucleophilicity *N* [29] indices, allow one to compare the reaction between different reactants and to assign the roles of electron donor and acceptor. The electrophilicity index has been successfully applied to 1,3-dipolar reactions [30]. Local indices are defined as the product of the global index of interest times the Fukui functions [31] for nucleo- and electro-philic attack *f^±^*. They measure the reactivity of molecular sites and allow to rank the isomeric reaction pathways in energy order thus providing selectivity prediction. Using *s* = *Sf^±^* [26] as reactivity index, one of us established a TS stabilisation energy criterion useful to predict the selectivity of cycloadditions [32]. This criterion has successfully been applied to several 1,3-dipolar cycloadditions [33,34,35,36], including reactions involving allenes [37] or azides [8,38]. Recently, Parr functions *P^±^* have been proposed and successfully applied to understand local reactivity, especially of reactions involving a polar TS [39]. We finally mention that recently the regioselectivity of electrophilic aromatic substitution reactions [40] and Diels-Alder cycloadditions [41] has been computationally studied by the local vibration mode approach [42].

The goal of the present article is to find out whether presently affordable computational schemes provide site- and regio-selectivity results for azide-allene cycloadditions in agreement with experimental results. The extent of this agreement will allow us to estimate how accurate predictions of site- and regio-selectivity can be made using computational chemistry.

**Figure 1:**
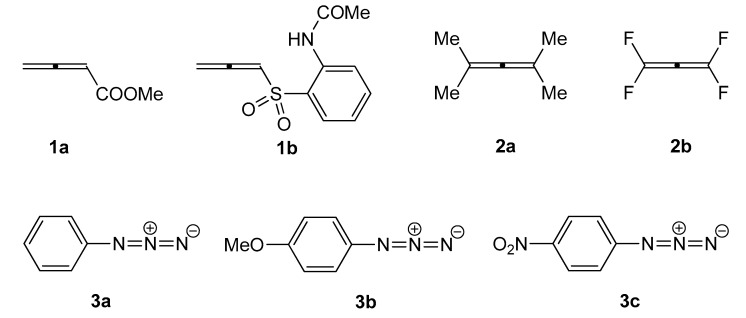
Mono- and tetra-substituted allenes to be submitted to cycloaddition with arylazides **3**.

In order to pursue our goal, the monosubstituted allenes **1a**,**b** and the tetrasubstituted ones **2a**,**b** were investigated (Figure 1). Experimental data on the behaviour of allenes **1a** [16] and **2a**,**b** [43,44] towards arylazides are available from the literature. Since these data are lacking for the known sulfonylallene **1b** [45], its cycloaddition with 4-methoxyphenylazide **3b** and 4-nitrophenylazide **3c** was realised experimentally for the first time in the present study.

The quantitative computational study of site- and regio-selectivity is very demanding because of the high energy accuracy required for meaningful predictions. Considering just two isomers produced under kinetic control, the isomer ratio is 1:9 when the reaction barriers differ by only 6.7 kJ/mol (1.6 kcal/mol) at 70 °C (a typical reaction temperature for azide-allene cycloadditions). Clearly, a quantitative prediction of the isomeric ratio requires an energy accuracy of ≈ 1 kJ/mol. Such an accuracy can only be achieved by high level computational protocols [46,47], which are too demanding to be applied to medium-sized molecules and to reaction in the condensed phase. Even though the best DFT functionals reproduce benchmark reaction barriers with accuracy in the 2.5–5.0 kJ/mol (0.6–1.2 kcal/mol) [48], the endeavour of reproducing isomeric ratios from DFT calculations may not be bound for failure. Indeed, one may argue that the calculation of isomer ratios benefits from systematic error cancellation as the reactants are the same for all isomers. The isomer ratio depends only on the difference between the energy of the transition state (TS) leading to the two isomers and inaccuracies in the calculation of the reactant energies do not affect the predicted isomer ratio. Furthermore, the isomeric TSs have similar geometric and electronic structure and, when medium- or large-size molecules are involved, the isomeric TSs may differ only in the relatively small molecular region where chemical bonds break and form. Therefore, it is not unlikely that the computational inaccuracies arising from the imperfect exchange-correlation functional, basis set incompleteness and superposition error, etc., are similar for the isomeric TSs and cancel each other to a significant extent when the difference of reaction barriers is taken.

We will calculate the activation barrier *differences* among the possible isomeric TSs using modern DFT functionals that proved effective and accurate for reaction barriers, [48] namely, M08-HX [49] and ωB97X-D [50]. The energy differences will be analysed within the framework of the distortion/interaction model [51]. Results will be compared to conventional B3LYP method. Furthermore, selectivity will be investigated using the conceptual DFT approach. As we will shortly show, the presently investigated reactions are expected to have nonpolar TSs. We will therefore use the TS stabilisation criterion based on local softness [32] to interpret the results.

## 2. Results and Discussion

### 2.1. Experimental

As far as monosubstituted allenes **1** are concerned, their intermolecular cycloaddition with arylazides **3** was performed by heating the appropriate mixture of the reagents in the absence of solvent. Allene **1b** was reacted with 4-methoxyphenylazide **3b** or 4-nitrophenylazide **3c** for 18 h at 65 °C, respectively, while the cycloaddition between allene **1a** and phenylazide **3a** required 24 h at 74 °C. [16] These harsh reaction conditions involved the unavoidable formation of some tarry material, on the other hand in the presence of a solvent the reaction did not proceed appreciably. This behaviour is not surprising because of the low reactivity of the allene moiety as dipolarophile even in the presence of appropriate activating substituents. [15] As an example, the tentative cycloaddition between allene **1b** and 4-methoxyphenylazide **3b** in carbon tetrachloride was accomplished for seven days at 40 °C showing that the starting reactants were almost unchanged, while prolonged heating in boiling carbon tetrachloride led to extensive decomposition of the starting azide.

**Scheme 1:**
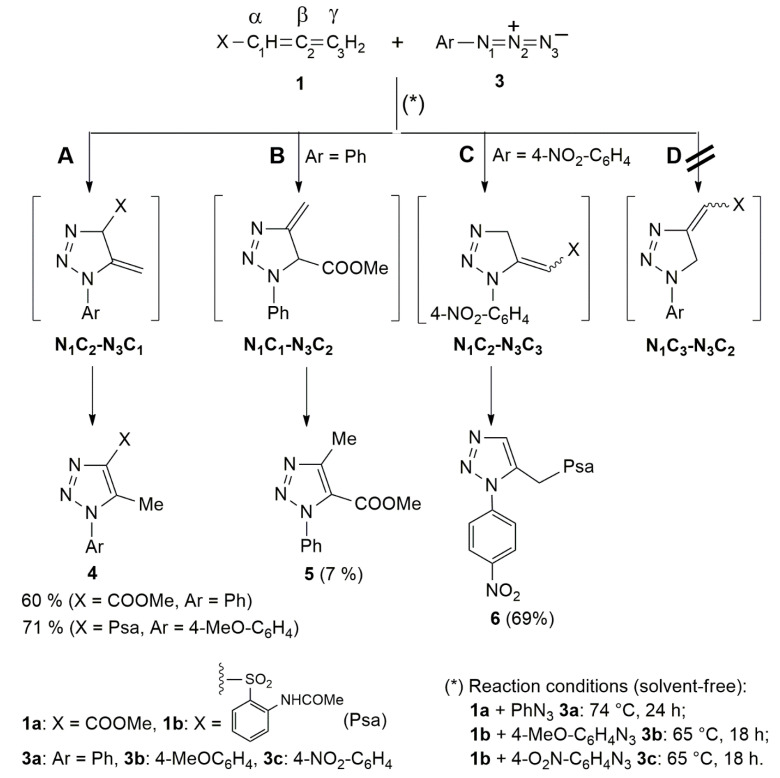
Cycloaddition between monosubstituted allenes **1** and arylazides **3**.

From the mechanistic point of view, the cycloaddition between monosubstituted allenes **1** and arylazides can follow the four pathways **A**–**D** outlined in the Scheme 1 via the four unstable primary cycloadducts arising from both allene α,β- and β,γ- double bonds. On the basis of experimental findings, in the reactions between arylazides and electron-deficient methoxycarbonylallene **1a** [16] and sulfonylallene **1b**, the route **D** can be safely ruled out.

It can be argued that the detection of the methylenetriazoline primary cycloadducts arising from the arylazide cycloadditions onto allenes **1a**,**b** may be accomplished at room temperature or below. In the case of methoxycarbonylallene **1a** it was impossible to envisage the intermediacy of such primary cycloadducts [16]. However, ^1^H-NMR evidence of the intermediacy of **N_1_C_2_-N_3_C_1_** was brought to light in the case of the sulfonylallene **1b** after standing for 24 h at 17 °C in a solution of 4-methoxyphenylazide in deuterated chloroform (see Supporting Information). However, the mentioned intermediate was elusive enough to prevent its isolation from the reaction mixture.

Finally, the formation of isomeric 1,2,3-triazole cycloadducts **4**–**6** should arise from a formal hydrogen 1,3-shift of the corresponding methylenetriazoline intermediates **N_1_C_2_-N_3_C_1_**, **N_1_C_1_-N_3_C_2_** and **N_1_C_2_-N_3_C_3_**. It is likely that this prototropic shift is facilitated by the acidity of the hydrogen in the 4- or 5-position of the above-mentioned intermediates.

The structures of novel cycloadducts **4b** and **6** were unambiguously determined through analytical and spectroscopic data. In particular, it was found useful to carry out NOESY experiments onto cycloadducts **4b** and **6**. In the former case, diagnostic NOE enhancement was found between the hydrogens of the methyl in the 5-position of the 1,2,3-triazole ring and the 2’,6’-aromatic hydrogens of the 4-methoxyphenyl ring. In the latter case, NOE enhancement was observed between the hydrogens of the methylene in the 5-position of the 1,2,3-triazole ring and the 2’,6’-aromatic hydrogens of the 4-nitrophenyl ring.

In the case of electron rich allenyl ethers, α,β site selectivity towards the allene moiety was observed [52]. However, this process will not be considered here since only the cycloaddition between allenyl ethers and the very dangerous picrylazide is known [52], making apparent the severe limitations of the reaction.

As can be envisaged from Scheme 1 and Table 1, the preferred cycloaddition orientation is that driving the substituted nitrogen of the dipole N_1_ to the central carbon of the allene C_2_; both cycloaddition site- and regio-selectivity issue cannot be rationalised on the grounds of FMO theory.

**Scheme 2:**
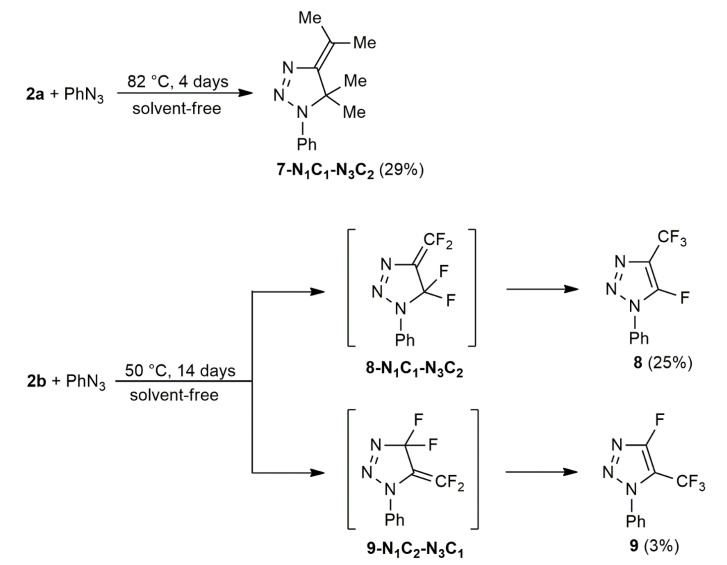
Cycloaddition between tetrasubstituted allenes **2** and phenylazide.

Tetramethylallene **2a** and tetrafluoroallene **2b** show quite different regioselectivities towards arylazide cycloaddition. Tetramethylallene **2a** reacts with phenylazide to give the cycloadduct **7-N_1_C_1_-N_3_C_2_** with 29% yield (Scheme 2) [43]. This means that the outer nitrogen of the dipole attacks the central carbon of **2a** with full selectivity (pathway **B**). Tetrafluoroallene **2b** reacts with phenylazide (at 50 °C for 14 days) [44] following a tandem cycloaddition-anionotropic rearrangement pathway to give a 89:11 mixture of triazoles **8** and **9** with 28% overall yield (Scheme 2). As for **2a**, the major isomer results from pathway **B**. The low conversion of both tetrasubstituted allenes **2** into the corresponding cycloadducts was due to the dimerisation of the allene via [2+2] cycloaddition processes.

### 2.2. Computational

#### 2.2.1. Global DFT Reactivity Indices

To get a preliminary insight into the mechanism and selectivity of the investigated azide-allene 1,3-DCs, we calculated some DFT global reactivity indices. In Table 2, we collected the electron chemical potential, µ, hardness, η, electrophilicity, ω, and nucleophilicity, *N*, of the reactants calculated at the M08-HX/pcseg-3//M08-HX/pcseg-2 and ωB97X-D/pcseg-3//ωB97X-D/pcseg-2 (the methods used below for the TS analysis). Since the electrophilicity and nucleophilicity scale were originally defined on the basis of B3LYP/6-31G(d,p) and B3LYP/6-31G(d) data, respectively, we also calculated the reactivity indices using these methods.

At the M08-HX/pcseg-3//M08-HX/pcseg-2 level, all allenes are moderate electrophiles, except for the marginal electrophile **2a**. **1b** and **2a** are moderate/strong nucleophiles while **1a** and **2b** are marginal nucleophiles. Clearly, **2a** will act as nucleophile, **1a** and **2b** as electrophile, and **1b** is reactive in both roles. Azides **3a** and **3b** are marginal electrophiles and strong nucleophiles while **3c** is a strong electrophile and a moderate/marginal nucleophile.

Comparing the values of µ, ω, and *N* for each reacting pair, it turns out that the considered 1,3-DCs have normal electron demand, i.e., electrons flow from the azide to the allene during the reaction, except for the **1b**+**3c** and **2a**+**3a** reactions. The azide-allene Δμ and Δω differences are small to moderate, ranging from −0.47 to 1.13 eV (Δμ) and from −0.32 to 0.57 eV (Δω). The **2a**+**3a** cycloaddition has the smallest Δμ = −0.16 eV (Δω = −0.10). Furthermore, being no allene a truly strong electrophile or nucleophile, in no case a strong electrophile reacts with a strong nucleophile. The transition states are then expected to have small polar character.

The ωB97X-D indices are very close to the M08-HX indices and of course lead to the same conclusions. The indices calculated following the original definition are somewhat different, as expected, since hybrid GGA B3LYP, hybrid meta-GGA M08-HX, and range-separated hybrid GGA ωB97X-D are quite different functionals. The fraction of exact exchange seems not relevant here as it is 52.23%, 20.0%, and 15.77% (short-range; it tends to 100% in the long range) for M08-HX, B3LYP, and ωB97X-D, respectively. However, analysis of the B3LYP indices also leads to the same qualitative conclusions regarding the nonpolar character of these 1,3-DCs. In view of these results, the following discussion of the conceptual DFT approach will be based on M08-HX indices only.

The global electron density transfer (GEDT) [54] was calculated at the M08-HX/pcseg-3//M08-HX/pcseg-2 level using natural population analysis (NPA, see SI).The GEDT at the TS are below 0.1 electrons for reactions involving **3a**, a value exceeded only by reactions involving substituted phenylazides and yielding isomer N_1_C_2_-N_3_C_1_. Comparison with the GEDT values at the TS of archetypal nonpolar (0.03 electrons) and polar (0.43 electrons) Diels-Alder reactions confirms that these cycloadditions can be classified as nonpolar reactions [55]. There is no correlation between the GEDT and the site- and regio-selectivity, showing that charge transfer is not predominant in determining the selectivity.

#### 2.2.2. Local DFT Reactivity Indices

Due to the nonpolar character of the TSs, the most appropriate local reactivity index is the local softness, as opposed to Parr functions suitable for polar TSs [39]. The local softness [56] was calculated as the global softness *S* = 1/η times the Yang and Mortier’s condensed Fukui functions *f* ^±^, calculated using the CHelp electrostatic atomic charges (Table 3). For normal electron demand reactions, *s*^+^ is appropriate for the allene and *s*^–^ for the azide; the reverse is true for the inverse demand 1,3-DCs.

In the symmetric tetrasubstituted allenes, the central C_2_ has the largest softness. The substituted C_1_ has the largest *s*^+^ in **1a**. In **1b**, the central C_2_ has the largest *s*^+^ while C_3_ has the largest *s*^–^. The local softness is much lower for **1b** than for the other allenes because the electron density changes upon addition/removal of electrons occur mainly in the ArSO_2_ moiety. As to the arylazides, the unsubstituted N_3_ atom has the largest *s*^+^ in both **3a** and **3c** while the largest *s*^–^ is found at N_3_ in **3a** and at N_1_ for **3b**. As the most reactive atom has the highest *s* values, one can surmise that in the major isomer a bond is formed between the atoms with the highest *s*. By inspection of Table 3 and taking into account the direction of the charge flow dictated by μ (Table 2), one can see that this criterion successfully indicates the major isomer in all cases.

In order to make quantitative predictions of the isomer ratios based on *s*, we consider the interaction grand potential ΔΩ between reactants. ΔΩ takes into account only charge transfer effects. It was calculated as in [32] and collected in Table 4. In this approach, the TS energy difference between isomers is proportional to corresponding ΔΩ difference. In the 1,3-DCs between **3a** and the symmetric allenes, ΔΩ is dominated by the N_3_-C_2_ term between the atoms with the largest atomic softness (see Table 3), irrespective of the opposite electron demands (see Table 2 and Table 3). Isomer N_1_C_1_-N_3_C_2_ has the most negative ΔΩ and is the major regioisomer, in agreement with experiment. The much less negative ΔΩ of N_1_C_2_-N_3_C_1_ agrees with the small fraction (or lack) of the minor isomer.

The 1,3-DC of arylazides to monosubstituted allenes may occur at both the unsubstituted and substituted double bond, in principle yielding four isomers. The ΔΩ of the four isomeric TSs of the **1a**+**3a** 1,3-DC is dominated by the large softness of C_1_ (see Table 3) leading to addition only to the substituted double bond, in agreement with experiment. The ΔΩ of N_1_C_2_-N_3_C_1_ is slightly larger than that of N_1_C_1_-N_3_C_2_ and therefore the former is the major isomer, and the latter is produced to a significant extent, again in agreement with experiment.

In the case of the reaction of **1b** with **3b** and **3c**, the lowest ΔΩ correctly indicates the major isomer. However, the ΔΩ of the isomeric TSs are similar, suggesting that several isomers may form in comparable quantity. This disagrees with the experiment, as just one isomer was isolated with both 4-substituted phenylazides. Therefore, the ΔΩ approach, which is based on the charge transfer between reactants just like the GEDT approach, is successful in pointing out the major isomer resulting from the cycloaddition of 4-substituted-phenylazides to allenes but predictions about minor isomers do not seem to be reliable.

#### 2.2.3. Transition States and Energetic Analysis

**Figure 2:**
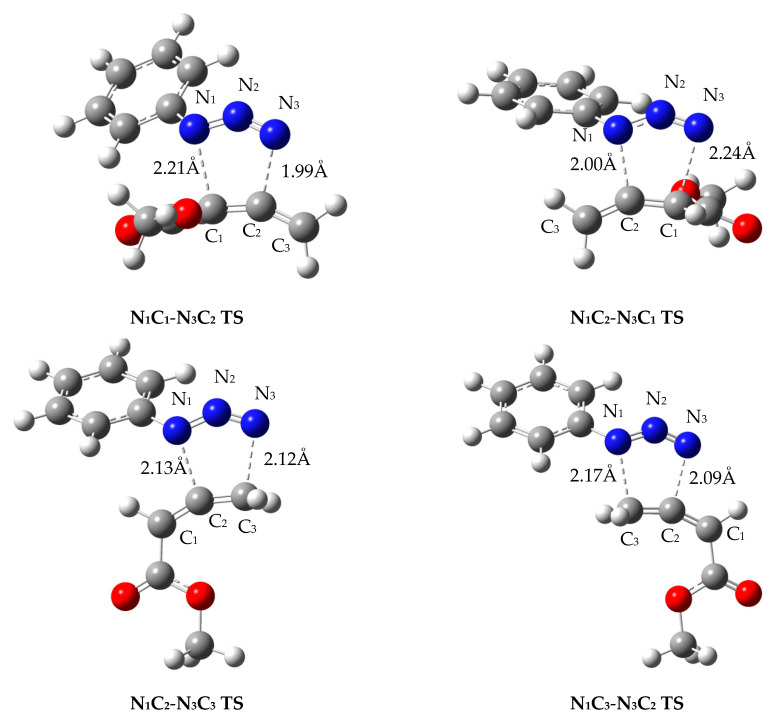
Structure of the transition states leading to the primary cycloadducts of the **1a**+**3a** cycloaddition calculated at the M08-HX/pcseg-2 level. Colour code: carbon, grey; nitrogen, blue; oxygen, red; hydrogen, light grey. The key atoms are numbered as in the text.

The geometries of all reactants, TSs, and products were optimised using the hybrid meta-GGA M08-HX functional and the triple-zeta pcseg-2 basis set. The TSs of the **1a**+**3a** 1,3-DC are depicted in Figure 2 and the TSs of the other investigated reactions can be found in the SI along with their Cartesian coordinates. In the harmonic analysis of the TSs, the eigenvector corresponding to the negative eigenvalue has in all cases large components corresponding to the approach of the two N-C atom pairs between which the new bonds form. To increase the computational accuracy, the electronic energy *E* of all systems was also calculated using the quadruple-zeta pcseg-3 basis set at the pcseg-2 geometry.

Some geometrical parameters of the TSs are collected in Table 5. The small difference between the lengths of the two C-N forming bonds (Δ) and the above-mentioned components of the imaginary-frequency eigenvector support the conclusion that the investigated 1,3-DCs occur via a one-step mechanism rather than a stepwise mechanism [57]. The major isomer is not related to the TS asymmetry, as measured by Δ = *R*_1_ – *R*_3_, nor to the early/late nature of the TS, as indicated by the average length <*R*> = (*R*_1_ + *R*_3_)/2 of the forming bonds (*R*_1_ is the distance from azide nitrogen N_1_ to the attacked carbon atom and similarly for *R*_3_).

It is interesting to relate the geometrical parameters of the TSs with the interaction grand potential ΔΩ (Table 4) and the decomposition of the TS electronic energy into the distortion and interaction contributions (Table 6) [51]. It is noteworthy that the bond making the most stabilising contribution to the interaction grand potential ΔΩ is longest in the major isomer, except for the **2a**+**3a** case, supporting the importance of charge transfer effects for the TS. If the latter effects were dominant, one would expect that *E*_int_ is indicative of the major isomer. This is so for the 1,3-DCs involving **1b** and **2a** but *E*_int_ points at the wrong isomer for **2b** and **1a**. This is further support to the conclusion that charge transfer effects are important but not dominating.

Therefore, we next consider distortion effects, as measured by the distortion energy *E*_dist_ and the azide N_1_-N̂_2_-N_3_ and allene C_1_-Ĉ_2_-C_3_ angles, which are both close to 180° in the reactants. The total *E*_dist_ indicates the correct major isomer for **1a**+**3a**, **2b**+**3a**, and **1b**+**3b**. Note that **1a**+**3a** and **2b**+**3a** are the cases that *E*_int_ fails to correctly indicate. For instance, the major isomer of **2b**+**3a** is determined by the favourable *E*_dist_ of the N_1_C_1_-N_3_C_2_ isomer that more than counteracts its unfavourable *E*_int_. We can conclude that both interaction and distortion effects are important to determine the selectivity, and which dominates depends on the particular reaction.

In the case of **1a**+**3a**, it is the favourable *E*_dist_ of **3a** that more than compensates both *E*_int_ and *E*_dist_ of **1a**. Actually, *E*_dist_ of the azide fragment in all cases is more negative for the major isomer, even when the global *E*_dist_ is not. Thus, it seems that the distortion energy of the azide fragment could be a reliable indicator of the major isomer. It would be interesting to check this finding on a broader scope of allene-azide reactions.

We now turn to the comparison of the experimental isomeric ratios to the calculated ones. The comparison is based on the following assumptions: (i) the transformation of the primary triazoline cycloadducts into the final triazoles is faster than the 1,3-DC between azide and allene, (ii) the latter is under kinetic control, and (iii) the rate constant *k* for a 1,3-DC can be written as

(1) $$k_{i}\;=\;A\;\exp(-\Delta H_{i}^{‡}/RT)$$

where *i* runs over all possible isomers, Δ*H*^‡^ is the activation enthalpy, *R* is the gas constant, *T* is the reaction temperature, and *A* is a prefactor that is equal for all isomeric TSs of any 1,3-DC. We use the activation enthalpy instead of the activation Gibbs energy because the thermochemistry is calculated using the standard expressions for an ideal gas in the rigid-rotor/harmonic-oscillator approximation. This leads to inaccuracy in the evaluation of the translational (and possibly rotational) activation entropy for solventless reactions such as the investigated ones. The relative amount of a particular isomer *j* can then be written as

(2) $$Y_{j}\;=\;\frac{k_{j}}{\sum_{i}k_{i}}\;=\;{\left[\underset{i}{\sum }e^{-\left(\Delta H_{i}^{‡}\;-\;\Delta H_{j}^{‡}\right)/RT}\right]}^{-1}\;=\;{\left[\underset{i}{\sum }e^{-\delta \Delta H_{ij}^{‡}/RT}\right]}^{-1}$$

Clearly, *Y_j_* does not depend on the enthalpy of the reactants since, being all *i* and *j* isomers, the reactant enthalpies cancel out:

(3) $$\delta \Delta H_{ij}^{‡}\;=\;\Delta H_{i}^{‡}\;-\;\Delta H_{j}^{‡}=\;H_{TS,i}\;-\;\underset{react}{\sum }H_{r}\;-\;H_{TS,j}\;+\;\underset{react}{\sum }H_{r}\;=\;H_{TS,i}\;-\;H_{TS,j}$$

The TS enthalpies *H*_TS_ were calculated as the sum of the electronic energy *E*(pcseg-3//pcseg-2) and enthalpic correction calculated with the pcseg-2 basis set. For the sake of comparison, we also calculated the isomeric ratios *Y* with the hybrid range-separated GGA functional ωB97X-D (including dispersion effects) and the popular hybrid GGA B3LYP. The TS enthalpy differences δΔ*H*^‡^ and the isomer ratios for each reaction are collected in Table 7 along with the experimental outcome. Detailed energetic data can be found in the SI, where it is also shown that isomeric ratios calculated using the activation Gibbs free energy are in worse agreement with experimental data than those calculated using Δ*H*^‡^. A picture of the energetics of the **1a+3a** cycloaddition can also be found in the SI.

In the case of **3a** reacting with the tetrasubstituted allenes, all functionals correctly indicate the major isomer. Quantitative agreement was obtained in the case of **2a**+**3a** with all functionals, when one takes into account that a few percent minor isomer can be lost during the isolation procedure. A slightly less satisfactory quantitative agreement is found for **2b**+**3a** for all functionals. In these two cases, a good qualitative and quantitative agreement is relatively easy to obtain since *Y* does not strongly depend on δΔ*H*^‡^ and all considered functionals achieve this goal.

In the **1a**+**3a** case, M08-HX and ωB97X-D correctly indicate the main site of attack (C_1_=C_2_) and the major isomer while B3LYP fails in both respects. Quantitatively, M08-HX and ωB97X-D gave very similar but not very satisfying results. The minor isomers are calculated to be too abundant with respect to the major isomer. Finally, considering the 1,3-DC between **1b** and the two 4-substituted phenylazides, M08-HX is able to provide correct indication of the major site and isomer for **1b**+**3b** but fails for **1b**+**3c**. On the other hand, the reliability of ωB97X-D functional is just reversed and accounts for the site selectivity of **1b**+**3c** only. B3LYP consistently predicts the wrong site of attack. From a quantitative standpoint, the results are not very satisfying. Even the best results, M08-HX with **1b**+**3b** and ωB97X-D with **1b**+**3c**, give too abundant minor isomers, as we have seen in the **1a**+**3a** case.

We can draw two conclusions from this analysis. First, hybrid functionals M08-HX and ωB97X-D, in conjunction with large basis-sets, are able to find the main site of attack and the main regioisomer in the azide-allene 1,3-DCs but they are not accurate enough to reproduce *Y* to within less than 10%. The former two perform similarly and in the following we shall use M08-HX, which has slightly smaller mean absolute and mean square deviation from the experimental *Y* across all investigated reactions. Second, M08-HX and ωB97X-D perform largely better than the hybrid GGA functional B3LYP in correctly ranking and quantitating the isomer yield. B3LYP should be abandoned in studies of reaction barriers.

Finally, we considered how to improve these results, i.e., what are the neglected factors that can decrease the accuracy of the DFT schemes. We first checked if any of the reactants/TSs/products have a significant multireference character impairing the single-reference Kohn-Sham wavefunction. The TAE(T) [46], *T*_1_ [58], and *D*_1_ [59] diagnostics were calculated for the **2b**+**3a** and **1b**+**3a** reactions (the latter as representative of both **1b**+**3b** and **1b**+**3c**). The multireference character is small in all cases and it is roughly constant on going from reactants to TS and product. Therefore, multireference does not seem to be of concern.

The incompleteness of the basis set was next considered though usually it does not significantly affect the energies of (partially) covalently bound molecular systems described by rather large basis sets. We investigated this effect by calculating the counterpoise (CP) correction at the M08-HX/pcseg-2 level. The CP correction to the TS electronic energy is always below 2 kJ/mol and the CP correction to the TS energy *difference* is <0.1 kJ/mol for the 1,3-DCs with the tetrasubstituted allenes, <0.4 kJ/mol for **1a**+**3a** and <1.0 kJ/mol for the 1,3-DCs to the arylsolfonylallene **1b**. The regioisomeric ratios are slightly affected and no improvement over the uncorrected results is obtained.

Computational treatment of the “solvent” effect in this case is not possible since these reactions are carried out by mixing the reactants, which are liquid at the reaction temperature, in the absence of solvent. To treat the liquid environment by the usual continuum models, several parameters are needed which are not known for our reactants. Based on rough chemical similarity, we carried out triple-zeta TS optimisation and thermochemistry and quadruple-zeta electronic energy calculations using the PCM model [60] for benzonitrile, which is a predefined solvent in the Gaussian suite. The calculated enthalpy differences and isomeric ratios are only slightly different from those obtained by calculations in vacuum (see SI).

## 3. Materials and Methods

Melting points were determined on a Büchi apparatus (Büchi, Flawil, CH) in open tubes and are uncorrected. IR spectra were recorded on a Perkin-Elmer 1725 X spectrophotometer (Perkin-Elmer, Waltham, MA, USA). Element analyses were taken on a Perkin-Elmer 2400, Series II CHNS/O Analyzer (Perkin-Elmer, Waltham, MA, USA). Mass spectra were determined on a VG-70EQ apparatus (Waters, Milford, MA, USA). ^1^H-NMR (300 MHz) and ^13^C-NMR (75 MHz) spectra were taken with a Bruker Fourier 300 instrument (in CDCl_3_ solutions at room temperature) (Bruker Corporation, Billerica, MA, USA). Chemical shifts are given as parts per million from tetramethylsilane. Coupling constants (*J*) values are given in hertz and are quoted to ±0.1 Hz consistently with NMR machine accuracy. NOESY experiments were performed by setting the following parameters: relaxation delay (d1) 2 s, irradiation power (dl2) 74 dB and total irradiation time (for each signal) 1.8 s.

The following compounds are known in the literature: methoxycarbonylallene **1a**: Ref. [16]; sulfonylallene **1b**: Ref. [45]; tetramethylallene **2a**: Ref. [43]; tetrafluoroallene **2b**: Ref. [44]; arylazides **3a**–**c**: Ref. [8].

### 3.1. Cycloaddition Between Allene ***1b*** and 4-Substituted-Phenyl Azides ***3b**,**c***

A mixture of sulfonylallene **1b** (237 mg, 1 mmol) and 4-methoxyphenylazide **3b** or 4-nitrophenylazide **3c** (1 mmol) was warmed at 65 °C for 18 h in the absence of solvent. The dark-brown residue was dissolved in chloroform (30 mL) and filtered on a silica gel-60 pad. The pale-yellow solution was evaporated at reduced pressure and the residue was chromatographed on a silica gel column with hexane-ethyl acetate 2:1. The eluate was evaporated at reduced pressure and the residue was crystallised with hexane-ethyl acetate affording pure cycloadducts **4b** or **6**.

1-(4-Methoxyphenyl)-4-[(2-acetylammino)phenylsulfonyl]-5-methyl-1*H*-1,2,3-triazole **4b**: (274 mg, 71%), m.p. 146–147 °C (dec.). ^1^H-NMR (CDCl_3_): 2.38 (3H, s, -COCH_3_), 2.47 (3H, s, triazole 5-CH_3_), 3.90 (3H, s, -OCH_3_), 7.05–8.46 (8H, m, aromatics), 9.59 δ (1H, br s, -NHCOMe). ^13^C-NMR (CDCl_3_): 9.4 (q, triazole 5-CH_3_), 25.2 (q, -NHCOCH_3_), 55.7 (q, -OCH_3_), 115.0 (d, aromatic C-H), 123.4 (d, aromatic C-H), 123.7 (d, aromatic C-H), 126.8 (d, aromatic C-H), 127.6 (s, phenyl C-N<), 128.7 (d, aromatic C-H), 135.3 (d, aromatic C-H), 137.0 (s, triazole C-4), 137.2 (s, phenyl C-SO_2_-), 143.8 (s, triazole C-5), 161.1 (s, -OCH_3_), 169.2 δ (s, -NHCOMe). IR (Nujol): 3380, 1710 cm^−1^; MS: *m/z*: 386 [M^+^]. HRMS (EI): *m*/*z*: [M^+^] calcd for C_18_H_18_N_4_O_4_S: 386.105, found 386.105. Elemental analysis calcd. (%) for C_18_H_18_N_4_O_4_S: C 55.95, H 4.70, N 14.50; found: C 55.88, H 4.73, N 14.42.

1-(4-Nitrophenyl)-5-[(2-acetylammino)phenylsulfonyl]methyl-1*H*-1,2,3-triazole **6**: (277 mg, 69%), m.p. 154–157 °C. ^1^H-NMR (CDCl_3_): 2.05 (3H, s, -COCH_3_), 4.71 (2H, s, triazole 5-CH_2_-), 7.18–8.40 (8H, m, aromatics), 9.37 δ (1H, br s, -NHCOMe). ^13^C-NMR (CDCl_3_): 25.0 (q, -NHCOCH_3_), 53.9 (t, triazole-5-CH_2_), 120.8 (d, aromatic C-H), 122.5 (d, aromatic C-H), 122.9 (d, aromatic C-H), 124.2 (d, aromatic C-H), 125.6 (d, aromatic C-H), 130.3 (d, aromatic C-H), 136.0 (d, triazole C_4_-H), 136.5 (s, phenyl C-SO_2_-), 138.0 (s, aromatic C-triazole N_1_), 140.6 (s, phenyl C-NO_2_), 147.6 (s, triazole C_5_), 168.4 δ (s, -NHCOMe). MS: *m/z*: 401 [M^+^]. Elemental analysis calcd. (%) for C_17_H_15_N_5_O_5_S: C 50.87, H 3.77, N 17.45; found: C 50.82, H 3.81, N 17.52.

### 3.2. Reaction Between Allene ***1b*** and 4-Methoxyphenylazide ***3b*** in Carbon Tetrachloride

A solution of sulfonylallene **1b** (237 mg, 1 mmol) and 4-methoxyphenylazide **3b** (149 mg, 1 mmol) in carbon tetrachloride (5 mL) was stirred at 17 °C for 170 h. Evaporation of the solvent gave a residue that was chromatographed on a silica gel column with hexane/ethyl acetate 3:2. The first fraction contained unreacted azide **3b** (112 mg, 75%). Further elution gave cycloadduct **4b** (23 mg, 6%) and unreacted sulfonylallene **1b** (171 mg, 72%).

### 3.3. Reaction Between Allene ***1b*** and 4-Methoxyphenylazide ***3b*** in CDCl_3_

A solution of sulfonylallene **1b** (47 mg, 0.2 mmol) and 4-methoxyphenylazide **3b** (30 mg, 0.2 mmol) in deuterated chloroform (1 mL) was stirred at 20 °C for 24 h. The solution was directly submitted to ^1^H-NMR analysis (see spectrum in the Supporting Information).

### 3.4. Computational Methods

All DFT calculations were carried out by the Gaussian09 [61] and Gaussian16 [62] program suites. We used Jensen pcseg-1, -2, and -3 basis sets [63] from the EMSL/PNNL Basis Set Exchange repository [64], and a (99,590) pruned integration grid (Int = UltraFine). DFT calculations were carried out using two semiempirical hybrid functionals, namely M08-HX, a global meta-GGA functional with 52.23% of exact exchange, and ωB97X-D, a long-range-corrected, range-separated hybrid GGA functional, which has 15.77% short-range exact exchange and tends to 100% exact exchange in the long range. For the sake of comparison, calculations using the semiempirical global hybrid GGA functional B3LYP, which includes 20.0% of exact exchange. The geometry of all reactants and TSs was fully optimised and characterised by harmonic analysis using the pcseg-2 basis set. All reactants have zero imaginary frequency and are energy minima. All TSs have a single imaginary frequency with eigenvector corresponding to the cycloaddition process. The thermal corrections to the electronic energy *E*, providing the thermodynamic functions *U*, *H*, *S* and *G,* were calculated in the rigid-rotor/harmonic-oscillator approximation at the experimental reaction temperature. The electronic energies of reactants and TSs were also calculated with the pcseg-3 basis set at the pcseg-2 geometry. The counterpoise correction to the electronic energy was calculated at the M08-HX/pcseg-3//M08-HX/pcseg-2 level. The calculations needed for the CP correction to BSSE provide the basis to analyse the site- and regio-isomeric reaction barriers within the framework of the distortion/interaction model [51]. Finally, the isomer ratios were calculated by assuming that (*i*) the reactions are under kinetic control, (*ii*) the rate constant can be written as *k* = *A* exp(−Δ*X*^‡^/*RT*) with *X* = *E*, *U*, *H*, and *G*, and (*iii*) the pre-exponential factor *A* is the same for all isomers.

The TAE(T) [46], T_1_ [58], and D_1_ diagnostics [59] were calculated at the CCSD(T) level using the VDZ(NP), VDZ(d), and VTZ(d) basis sets at the M08-HX/pcseg-2 geometry.

The DFT global reactivity indices were calculated at the M08-HX/pcseg-3//M08-HX/pcseg-2 level using the energy of the neutral reactant *E*(*n*) and of its radical anion *E*(*n* + 1) and cation *E*(*n* – 1) in the rigid approximation, thus allowing for electron density relaxation (*n* is the number of electrons). Electron chemical potential (µ), hardness (η), softness (*S*), and electrophilicity (ω) were calculated via the ionisation potential IP = *E*(*n* − 1) – *E*(*n*) and electron affinity EA = *E*(*n*) − *E*(*n* + 1) as follows [65]:

μ = (IP + EA)/2(4)

η = IP − EA(5)

*S* = (IP + EA)^–1^ (6)

ω = μ^2^*S*/2 (7)

The nucleophilicity index *N* is here computed as

*N* = IP − IP(TCE) (8)

where IP(TCE) is the IP of tetracyanoethene. For the sake of consistency with the original definitions [28,53], ω and *N* were also computed at the B3LYP/6-31G(d,p) and B3LYP/6-31G(d) level, respectively, with the approximations IP ≈ –ε_HOMO_ and EA ≈ –ε_LUMO_.

The Fukui functions *f^±^* for nucleophilic and electrophilic attack to atom *k* were calculated from the electrostatic CHelp [66] charges *q_k_* of the neutral system and its radical ions, calculated at the M08-HX/pcseg-3//M08-HX/pcseg-2 level, as:

*f*^+^ = −[*q_k_*(*N* + 1) − *q_k_*(*N*)] (9)

*f*^–^ = −[*q_k_*(*N* − 1) − *q_k_*(*N*)] (10)

Electrostatic charges have already proved suitable to the calculation of atomic Fukui functions [8,33,35]. The local softness condensed to atom *k* was calculated from:

(11) $$s_{k}^{\pm }=S\;f_{k}^{\pm }$$

Finally, the energy stabilisation $\Delta \Omega_{ij}^{kl}$ of the TS with new bonds forming between atoms N*_i_* and C*_k_* and between atoms N*_j_* and C*_l_* was calculated as [32]:

(12) $$\Delta \Omega_{ij}^{kl}\;=\;\Delta \Omega_{i}^{k}+\Delta \Omega_{j}^{l}$$

(13) $$\Delta \Omega_{i}^{k}\;=\;-\frac{1}{2}{\left(\mu_{\mathrm{azide}}-\mu_{\mathrm{allene}}\right)}^{2}\frac{s_{i}s_{k}}{s_{i}+s_{k}}$$

where the appropriate local softness *s*^+^ or *s*^–^ must be used in relation to the role of electron-donor or -acceptor of the corresponding reactant.

The global electron density transfer (GEDT) [54] at the TS was calculated partitioning the atomic natural population [67] between the azide and allene fragments.

## 4. Conclusions

The construction of highly substituted 1,2,3-triazoles represent a valuable target of contemporary organic synthesis since their well-known utility in both academic and practical perspectives. In particular, the 1,3-dipolar cycloaddition between azides and allenes suggests a useful method for the synthesis of highly functionalised 1,2,3-triazoles that are difficult to obtain by other routes. The site- and regio-chemical control of these reactions are generally satisfactory, and it is quite interesting to investigate it beyond the simple "maximal HOMO-LUMO overlap" rule since the results obtained within the FMO theory are sometimes misleading. We applied both DFT approaches (conceptual and computational) to this problem, using semiempirical hybrid functionals, with high fraction of exact exchange, and DFT-optimised triple and quadruple-zeta basis sets. We could establish that the azide-allene 1,3-DC has a one-step, nonpolar TS and that both distortion (nuclear potential) and interaction (charge transfer) energies significantly contribute to determine both site- and regio-selectivity. The conceptual ΔΩ approach provides correct indication of the major isomer. The TS enthalpies computed with the M08-HX and ωB97X-D provide a semiquantitatively correct description of the selectivity. The major drawback of the present computational approach probably is the lack of affordable methods to describe a reaction carried out without solvent. Molecular dynamics simulations seem to represent a viable approach to this problem, provided one can afford their high computational cost.

## Figures and Tables

**Table 1:** Cycloaddition between allenes **1** and **2** and arylazides **3**.

Entry	Reaction	Overall Yield	Product Ratio	
		(%)	**4:5**	**8:9**
1	**1a**+**3a**	67	90:10	-
2	**1b**+**3b**	71	100:0	-
3	**1b**+**3c**	69 ^a^	-	-
4	**2a**+**3a**	29 ^b^	-	-
5	**2b**+**3a**	28	-	90:10

^a^ Cycloadduct **6-N_1_C_2_-N_3_C_3_** was obtained as the unique regioisomer. ^b^ Cycloadduct **7-N_1_C_1_-N_3_C_2_** was obtained as the unique regioisomer.

**Table 2:** Electron chemical potential (µ), hardness (η), softness (*S*), electrophilicity (ω), and nucleophilicity (*N*) of the investigated allenes and azides. All quantities are in given in eV.

	B3LYP/6-31G(d,p)					M08-HX/pcseg-3 //M08-HX/pcseg-2					ωB97X-D/pcseg-3 //ωB97X-D/pcseg-2			
	µ	*S*	ω	*N* ^a^		µ	*S*	ω	*N*		µ	*S*	ω	*N*
**2a**	−2.55	0.137	0.44	2.91		−3.73	0.098	0.68	3.14		−3.42	0.098	0.57	3.13
**2b**	−4.18	0.136	1.19	1.27		−5.02	0.079	1.00	0.66		−4.62	0.080	0.85	0.77
**1a**	−4.13	0.163	1.39	1.92		−4.91	0.091	1.10	1.59		−4.65	0.093	1.00	1.60
**1b**	−4.08	0.199	1.66	2.54		−4.77	0.116	1.32	2.90		−4.53	0.116	1.19	2.78
**3a**	−3.63	0.193	1.27	2.92		−3.89	0.103	0.78	3.23		−3.66	0.103	0.69	3.13
**3b**	−3.29	0.210	1.13	3.46		−3.66	0.112	0.75	3.85		−3.46	0.113	0.68	3.76
**3c**	−4.82	0.229	2.66	2.12		−5.24	0.119	1.64	2.54		−5.03	0.121	1.53	2.47

^a^ Calculated with the 6-31G(d) basis set, following the definition in [53].

**Table 3:** Atoms with the largest local softness *s* (eV^−1^), calculated at the M08-HX/pcseg-3//M08-HX/pcseg-2 level, within the investigated allenes and azides.

Compound	Atom	1000 *s* (eV^−1^)
**2a**	C_2_	23.1 (*s*^–^)
**2b**	C_2_	50.5 (*s*^+^)
**1a**	C_1_	20.9 (*s*^+^)
**1b**	C_2_	5.3 (*s*^+^)
**1b**	C_3_	1.3 (*s*^–^)
**3a**	N_3_	20.9 (*s*^+^)
**3a**	N_3_	24.4 (*s*^–^)
**3b**	N_1_	23.1 (*s*^–^)
**3c**	N_3_	20.2 (*s*^+^)

**Table 4:** Interaction grand potential ΔΩ (kJ/mol) at the transition state of the investigated cycloadditions, calculated at the M08-HX/pcseg-3//M08-HX/pcseg-2 level. The bond contributions are reported in brackets, ordered as in the column headings.

	ΔΩ (kJ/mol)			
Reaction	N_1_C_2_-N_3_C_1_	N_1_C_2_-N_3_C_1_	N_1_C_2_-N_3_C_3_	N_1_C_3_-N_3_C_2_
**2a**+**3a**	−0.22 (−0.05, −0.17)	−0.05 (−0.05,0.00)	–	–
**2b**+**3a**	−0.91 (−0.20, −0.71)	−0.05 (−0.17,0.12)	–	–
**1a**+**3a**	−0.51 (−0.48, −0.04)	−0.60 (−0.04, −0.56)	0.30 (−0.04,0.34)	0.34 (0.38, −0.04)
**1b**+**3b**	−0.34 (-0.10, −0.24)	−0.35 (−0.26, −0.10)	−0.29 (−0.26, −0.04)	−0.28 (−0.04, −0.24)
**1b**+**3c**	−0.019(−0.007, −0.012)	−0.018(−0.011, −0.007)	−0.025 (−0.011, −0.013)	−0.024 (−0.012, −0.012)

**Table 5:** Selected geometrical parameters of the TSs calculated at the M08-HX/pcseg-2level.

Reaction	Isomer	*R*_1_^a^ (Å)	*R*_3_^b^ (Å)	<*R*> ^c^ (Å)	Δ ^d^ (Å)	N_1_-N̂_2_-N_3_ (°)	C_1_-Ĉ_2_-C_3_ (°)
**2a**+**3a**	N_1_C_1_-N_3_C_2_	2.33	2.12	2.22	0.21	139.9	147.3
	N_1_C_2_-N_3_C_1_	2.17	2.20	2.18	–0.03	137.1	151.5
**2b**+**3a**	N_1_C_1_-N_3_C_2_	2.12	2.15	2.14	–0.03	141.0	146.3
	N_1_C_2_-N_3_C_1_	2.12	2.06	2.09	0.07	140.2	145.4
**1a**+**3a**	N_1_C_1_-N_3_C_2_	2.21	1.99	2.10	0.22	139.2	151.8
	N_1_C_2_-N_3_C_1_	2.00	2.24	2.12	–0.24	140.9	149.8
	N_1_C_2_-N_3_C_3_	2.13	2.12	2.13	0.01	139.2	154.9
	N_1_C_3_-N_3_C_2_	2.17	2.09	2.13	0.08	139.5	154.4
**1b**+**3b**	N_1_C_1_-N_3_C_2_	2.14	2.09	2.11	0.05	140.1	150.4
	N_1_C_2_-N_3_C_1_	2.15	2.13	2.14	0.03	143.2	146.3
	N_1_C_2_-N_3_C_3_	2.23	1.94	2.09	0.29	140.2	152.3
	N_1_C_3_-N_3_C_2_	1.92	2.33	2.12	–0.41	140.5	153.9
**1b**+**3c**	N_1_C_1_-N_3_C_2_	2.13	2.13	2.13	0.00	140.3	153.9
	N_1_C_2_-N_3_C_1_	2.21	1.95	2.08	0.26	138.7	151.0
	N_1_C_2_-N_3_C_3_	1.95	2.27	2.11	–0.33	141.7	147.3
	N_1_C_3_-N_3_C_2_	2.14	2.09	2.11	0.05	139.2	152.0

^a^ Length of the bond between N_1_ and the attacked carbon atom. ^b^ Length of the bond between N_3_ and the attacked carbon atom. ^c^ Average length of the forming bonds, (*R*_1_ + *R*_3_)/2. ^d^ Length difference of the forming bonds, *R*_1_–*R*_3_.

**Table 6:** Energy decomposition of the isomeric TSs calculated using the M08-HX functionals. The interaction and distortion energies are given relative to the major isomer so that a positive δ*E* is an unfavourable energetic contribution.

			δ*E*_dist_ (kJ/mol)		
Reaction	Isomer	δ*E*_int_ (kJ/mol)	Total	Azide	Allene
**2a**+**3a**	N_1_C_1_-N_3_C_2_	0	0	0	0
	N_1_C_2_-N_3_C_1_	4.4	4.8	11.6	–6.8
**2b**+**3a**	N_1_C_1_-N_3_C_2_	0	0	0	0
	N_1_C_2_-N_3_C_1_	–2.7	27.7	22.5	5.3
**1a**+**3a**	N_1_C_1_-N_3_C_2_	–1.1	2.5	9.8	–7.3
	N_1_C_2_-N_3_C_1_	0	0	0	0
	N_1_C_2_-N_3_C_3_	11.9	–8.1	3.8	–11.9
	N_1_C_3_-N_3_C_2_	15.6	–10.2	3.8	–13.9
**1b**+**3b**	N_1_C_1_-N_3_C_2_	13.4	1.2	18.0	–16.8
	N_1_C_2_-N_3_C_1_	0	0	0	0
	N_1_C_2_-N_3_C_3_	25.4	–22.4	12.7	–35.1
	N_1_C_3_-N_3_C_2_	43.0	–39.0	9.9	–49.0
**1b**+**3c**	N_1_C_1_-N_3_C_2_	15.5	–1.2	13.8	–15.0
	N_1_C_2_-N_3_C_1_	0	0	0	0
	N_1_C_2_-N_3_C_3_	24.9	–23.0	7.2	–30.2
	N_1_C_3_-N_3_C_2_	46.1	–44.9	2.5	–47.3

**Table 7:** Enthalpy difference between isomeric TSs (δΔ*H*^‡^) and corresponding isomeric ratio (*Y*), calculated using the M08-HX, ωB97X-D, and B3LYP functionals and compared to the experimental isomeric ratio.

			M08-HX			ωB97X-D			B3LYP			Exp
Reaction	Isomer		δΔ*H*^‡^ (kJ/mol)	*Y* (%)		δΔ*H*^‡^ (kJ/mol)	*Y* (%)		δΔ*H*^‡^ (kJ/mol)	*Y* (%)		*Y* (%)
**2a**+**3a**	N_1_C_1_-N_3_C_2_		0	96		0	97		0.0	99		100
	N_1_C_2_-N_3_C_1_		9.5	4		10.0	3		13.7	1		0
**2b**+**3a**	N_1_C_1_-N_3_C_2_		0	100		0.0	100		0.0	100		89
	N_1_C_2_-N_3_C_1_		24.9	0		22.9	0		22.3	0		11
**1a**+**3a**	N_1_C_1_-N_3_C_2_		2.0	27		2.4	24		1.6	24		10
	N_1_C_2_-N_3_C_1_		0.0	54		0	56		3.6	12		90
	N_1_C_2_-N_3_C_3_		4.1	13		4.1	13		2.0	21		0
	N_1_C_3_-N_3_C_2_		6.1	7		6.5	6		0	43		0
**1b**+**3b**	N_1_C_1_-N_3_C_2_		15.7	0		19.5	0		19.7	0		0
	N_1_C_2_-N_3_C_1_		0	66		3.9	17		19.4	0		100
	N_1_C_2_-N_3_C_3_		2.8	25		0	69		6.0	10		0
	N_1_C_3_-N_3_C_2_		5.6	9		4.4	14		0	90		0
**1b**+**3c**	N_1_C_1_-N_3_C_2_		15.4	0		19.6	0		23.5	0		0
	N_1_C_2_-N_3_C_1_		0	51		5.4	8		28.1	0		0
	N_1_C_2_-N_3_C_3_		1.6	28		0	58		8.8	4		100
	N_1_C_3_-N_3_C_2_		2.6	20		1.5	34		0	96		0

## Data Availability

The data presented in this study are available in this article.

## Supplementary Materials

The following are available online, spectroscopic and spectrometric data of novel 1,2,3-triazoles **4b,c** 1H-NMR spectrum of the intermediate N1C2-N3C1 (X = Psa, Ar = 4-MeOC_6_H_4_). Structure of all TSs calculated at the M08-HX/pcseg-2 level. Energetics and isomer ratios of all TSs calculated with different functionals and the pcseg-2 basis set.
