# Supplementary material for: The Azide-Allene Dipolar Cycloaddition: Is DFT Able to Predict Site- and Regio-Selectivity?
Source: Molecules. 2021 Feb 10;26(4):928. doi: 10.3390/molecules26040928 (PMC7916341; doi:10.3390/molecules26040928)
Supplement: Supplementary file 1 [file molecules-26-00928-s001.pdf]

Supplementary Materials

The azide-allene dipolar cycloaddition: Is DFT able to predict site- and regio-selectivity?

Giorgio Molteni and Alessandro Ponti

Summary

^{1}H and ^{13}C NMR spectra of cycloadduct **4b** and intermediate **N_1C_2-N_3C_1-b**; NOESY, IR, and Mass spectrum of cycloadduct **4b** and **6** 2

Global electron density transfer (M08-HX/pcseg-3//M08-HX/pcseg-2) 8

Structure of the transition states (M08-HX/pcseg-2) 9

Cartesian coordinates of the transition states (M08-HX/pcseg-2) 13

Energetics of the transition states 29

^{1}H and ^{13}C NMR spectra of cycloadduct **4b** and intermediate N_1C_2-N_3C_1-b; NOESY, IR, and Mass spectrum of cycloadduct **4b** and **6**

(*A*) ^{1}H NMR spectrum of cycloadduct **4b**

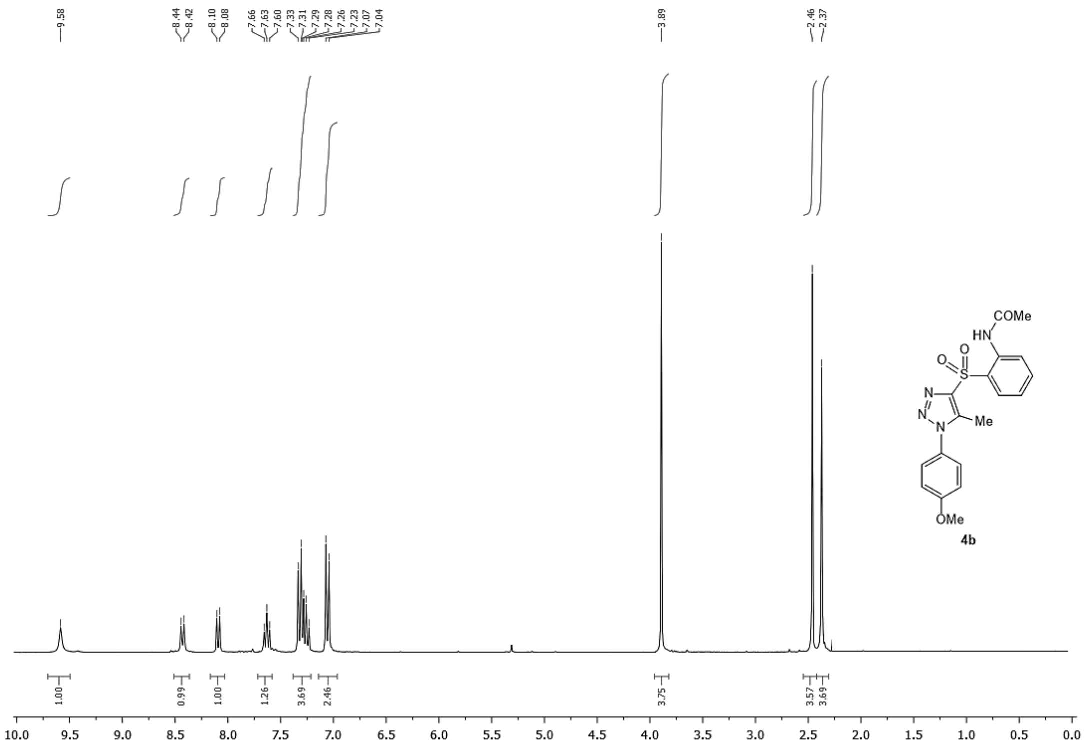

(*B*) ^{13}C NMR spectrum of cycloadduct **4b**

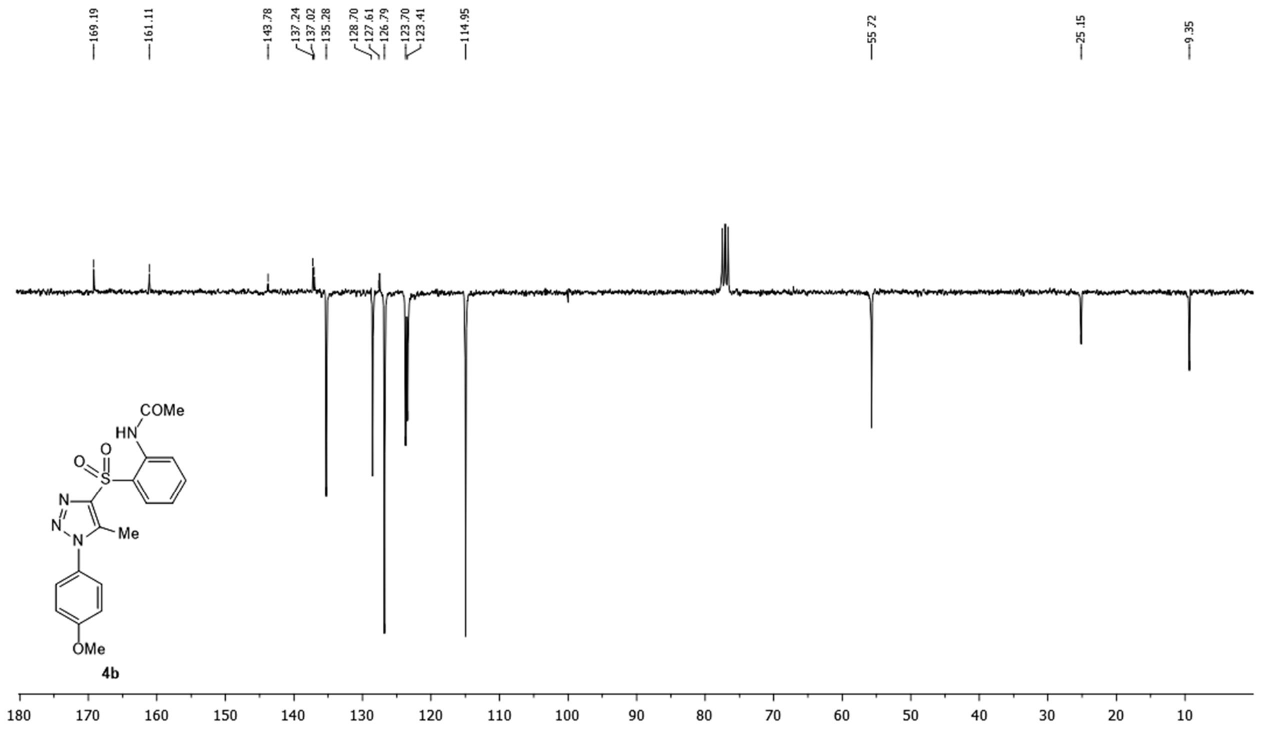

(*C*) NOESY spectrum of cycloadduct **4b**

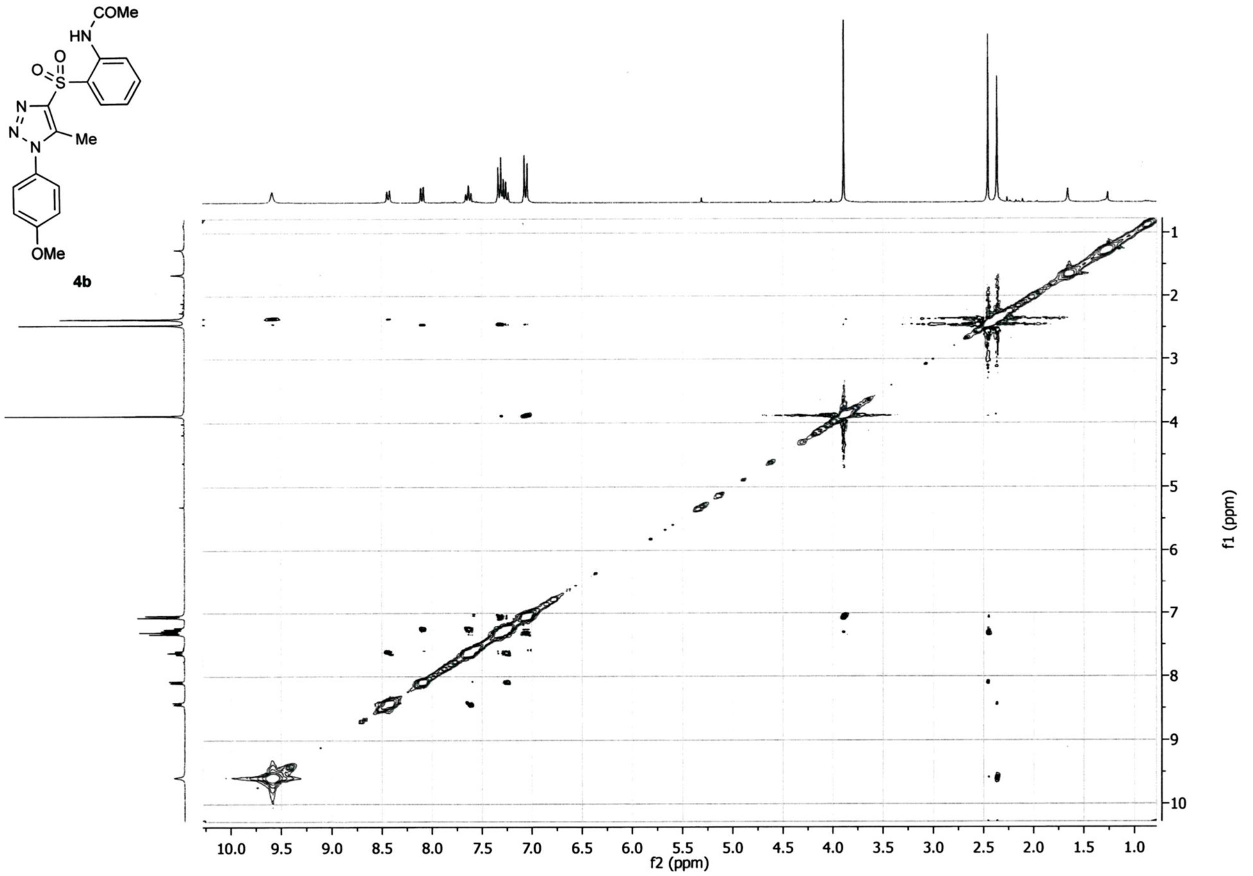

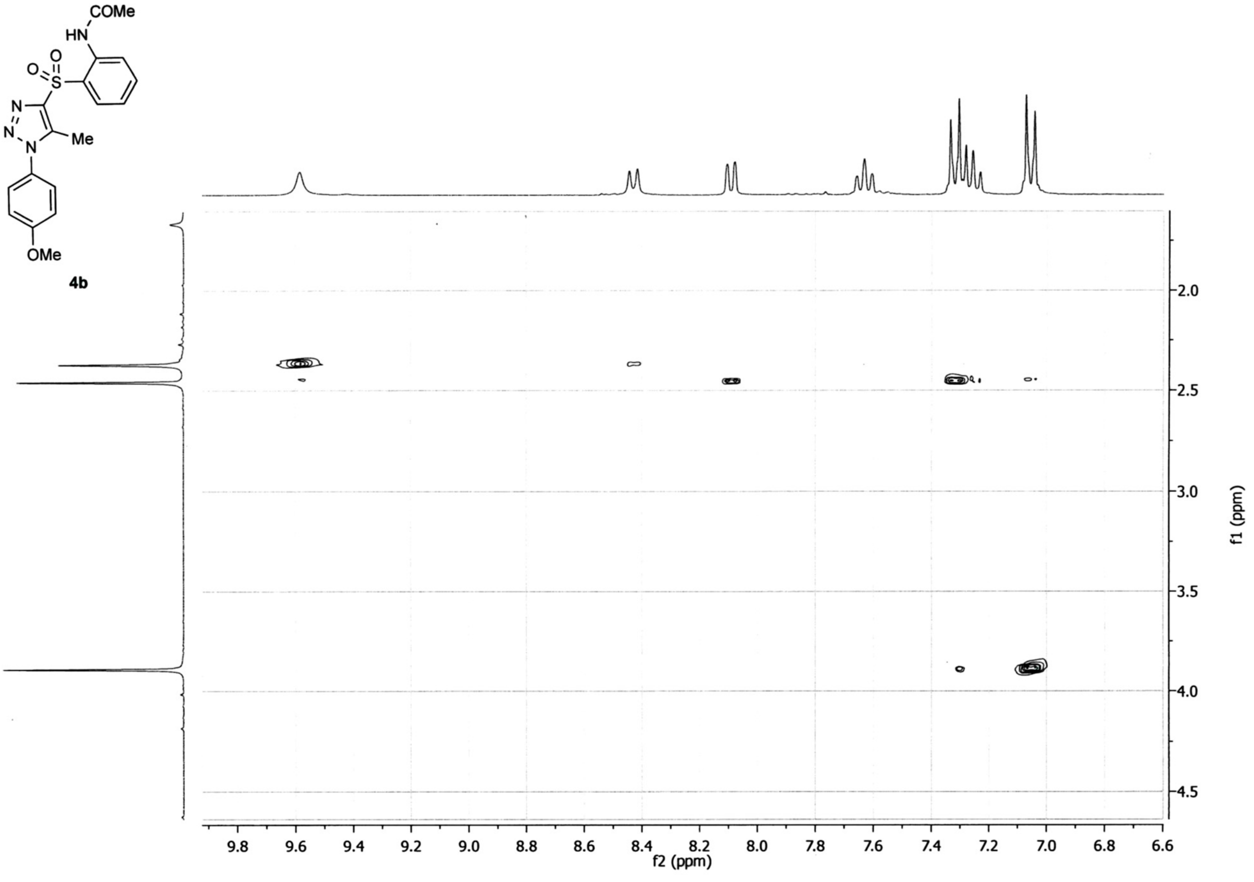

(*D*) IR spectrum of cycloadduct **4b**

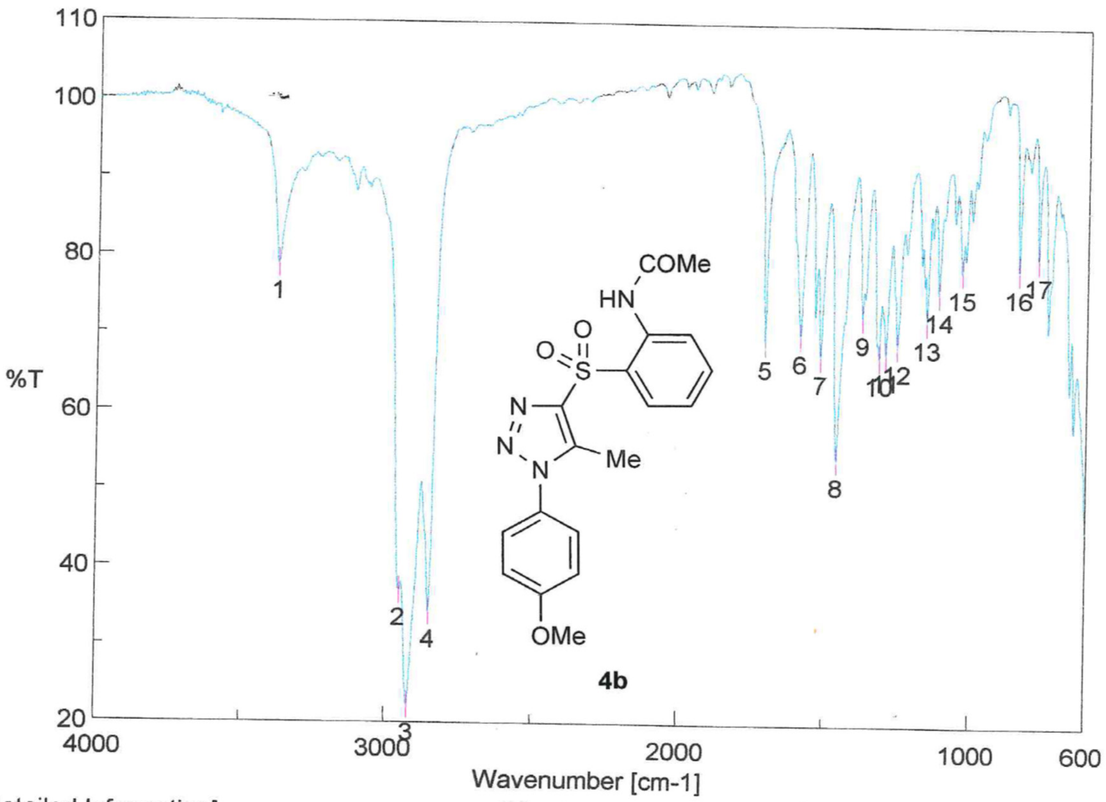

[Detailed Information]

Creation date	01/07/2019 10:27
Data array type	Linear data array
Horizontal axis	Wavenumber [cm-1]
Vertical axis	%T
Start	599.753 cm-1
End	4000.6 cm-1
Data interval	0.964233 cm-1
Data points	3528

[Measurement Information]

Model Name	FT/IR-4100typeA
Serial Number	B005261016
Measurement Date	01/07/2019 10:27
Light Source	Standard
Detector	TGS
Accumulation	16
Resolution	4 cm-1
Zero Filling	On
Apodization	Cosine
Gain	Auto (2)
Aperture	Auto (7.1 mm)
Scanning Speed	Auto (2 mm/sec)
Filter	Auto (30000 Hz)

[Result of Peak Picking]

No.	Position	Intensity	No.	Position	Intensity
1	3378.67	78.796	2	2953.45	36.922
3	2923.56	22.1857	4	2853.17	34.1784
5	1708.62	69.2778	6	1589.06	70.1193
7	1518.67	67.4919	8	1460.81	54.2476
9	1375.96	72.5869	10	1317.14	67.4372
11	1294.97	67.8553	12	1255.43	68.9795
13	1153.22	72.0539	14	1112.73	75.6574
15	1034.62	78.5489	16	837.919	78.672
17	770.423	80.3073			

(*E*) Mass spectrum of cycloadduct **4b**

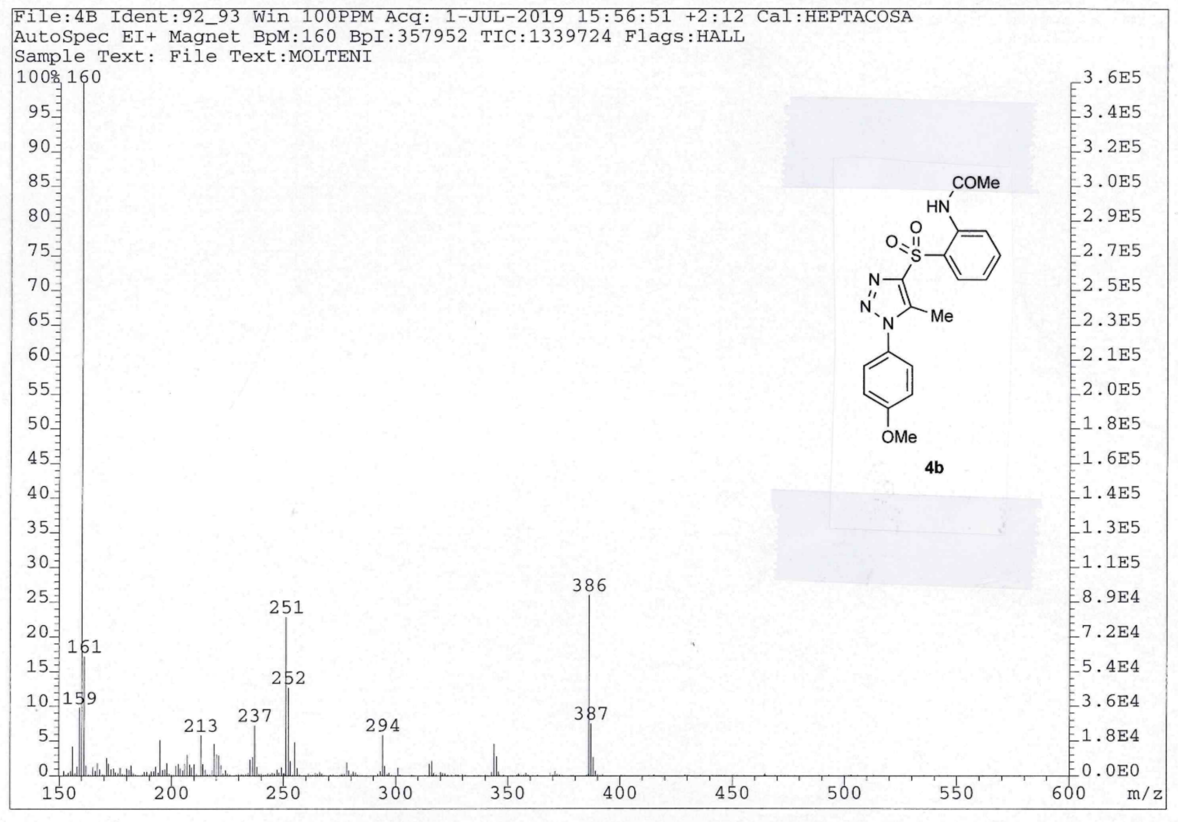

(*F*) High-resolution mass spectrum of cycloadduct **4b**

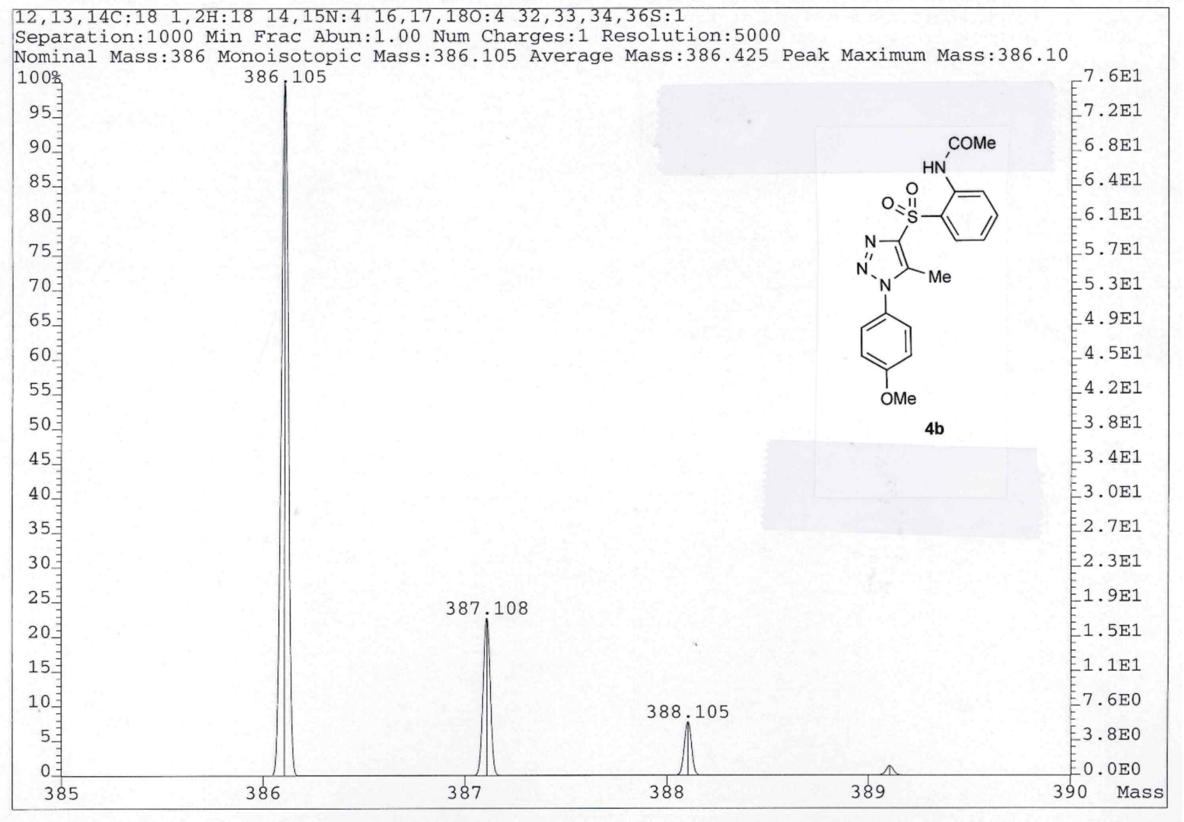

(*G*) ^{1}H NMR spectrum of cycloadduct **6**

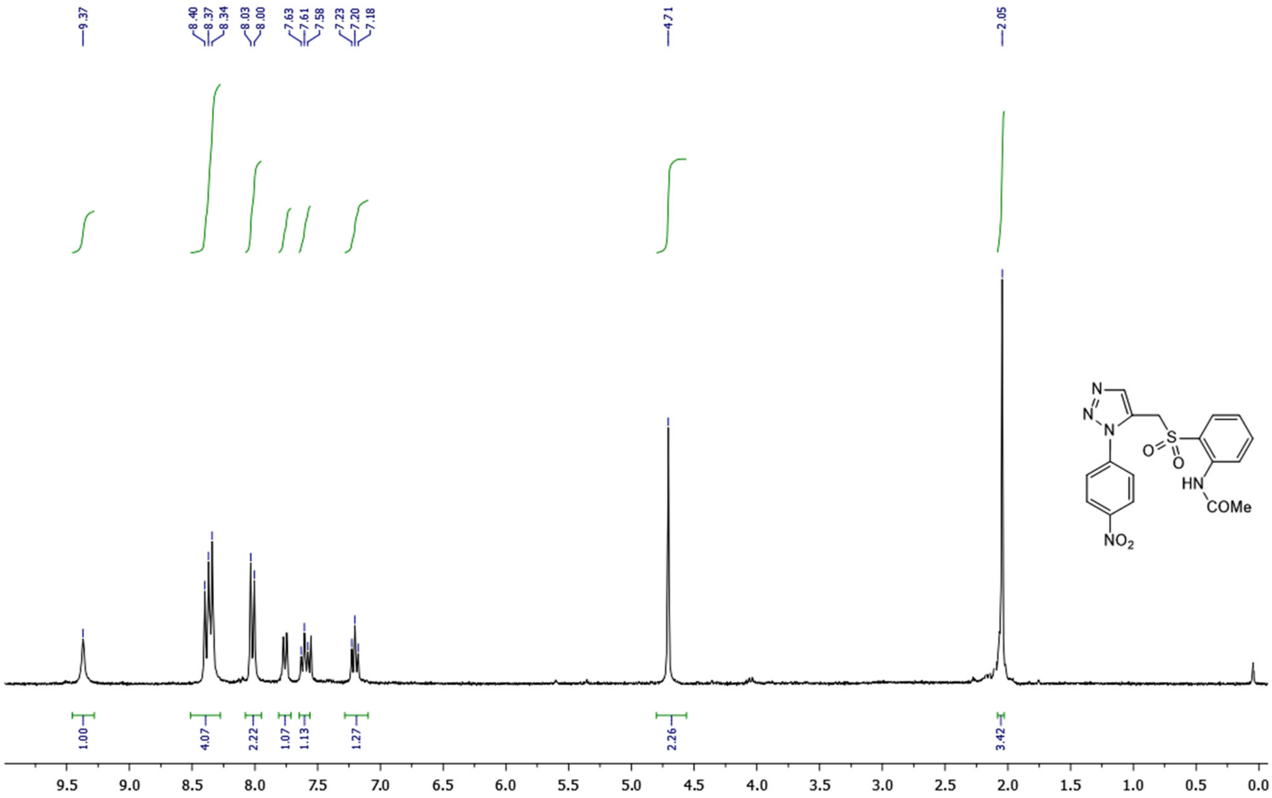

(*H*) ^{13}C NMR spectrum of cycloadduct **6**

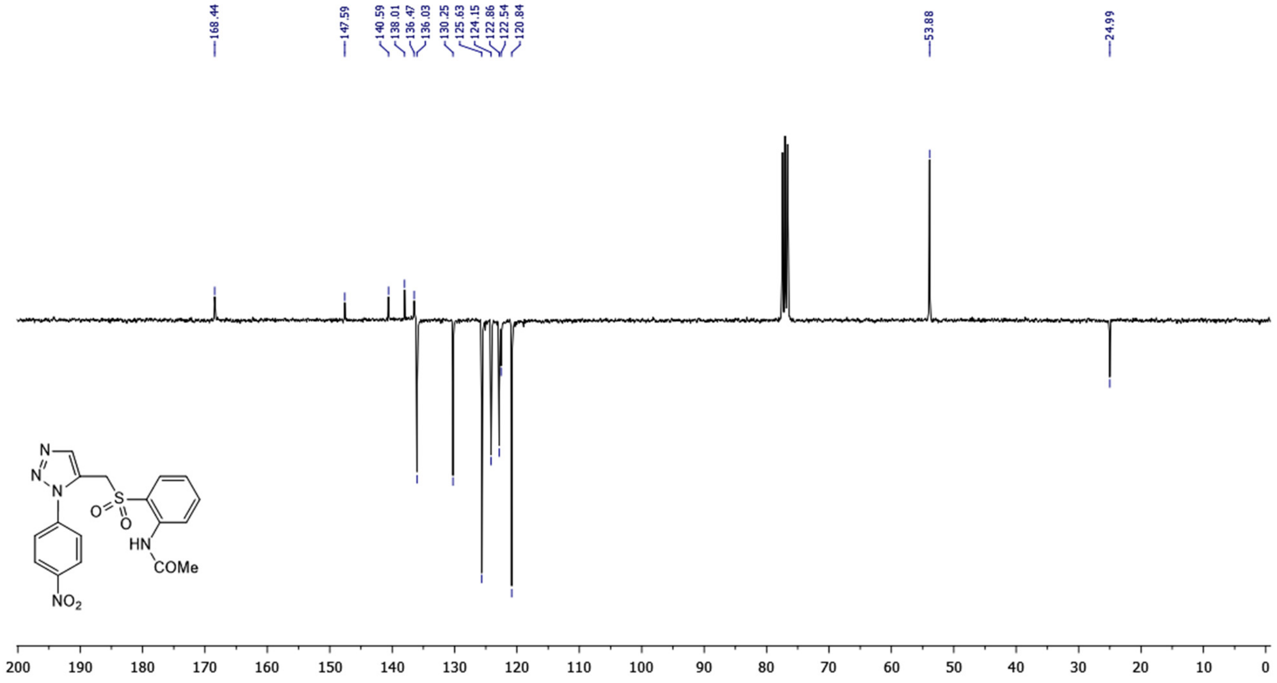

(*I*) NOESY spectrum of cycloadduct **4b**

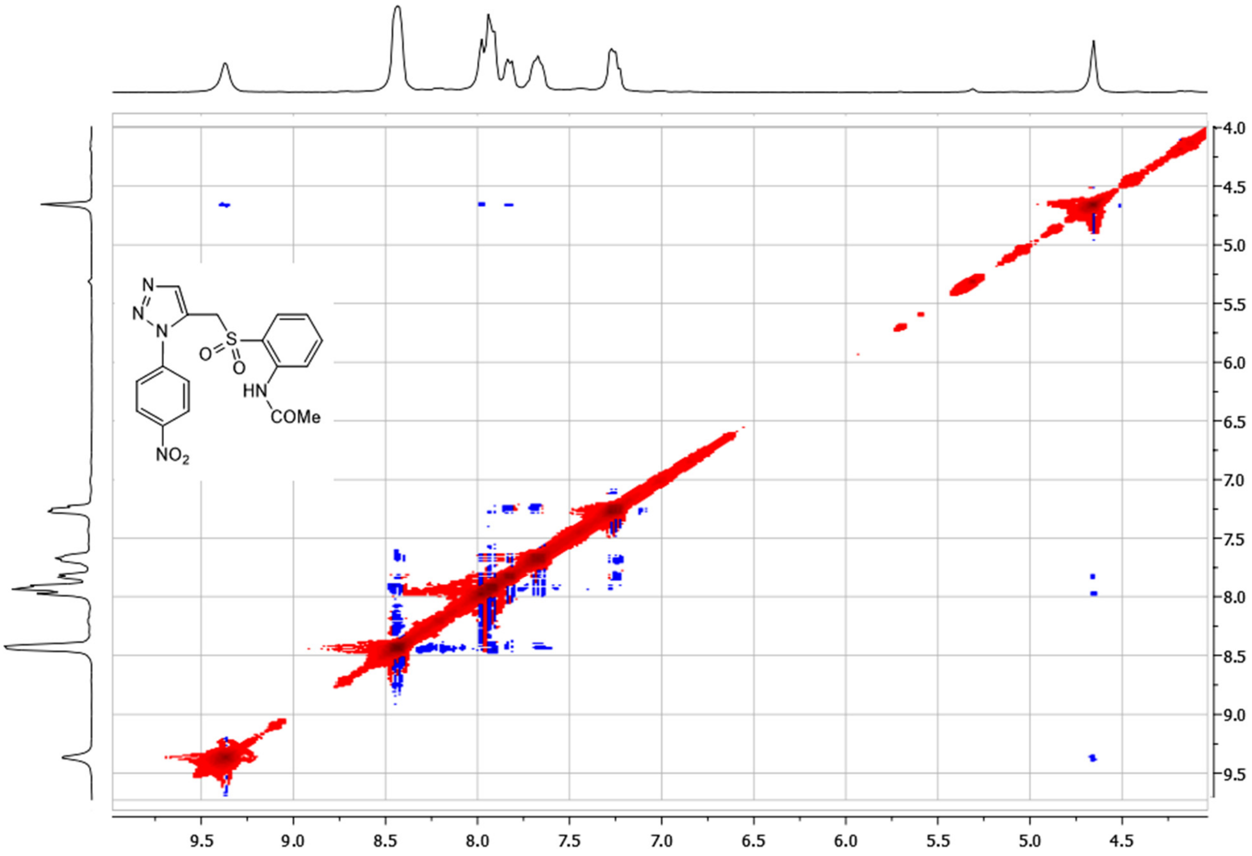

(*J*) ^{1}H NMR spectrum of the intermediate **N_1C_2-N_3C_1-b** (plus starting reagents: sulfonylallene **1b** and 4-methoxyphenylazide **3b**).

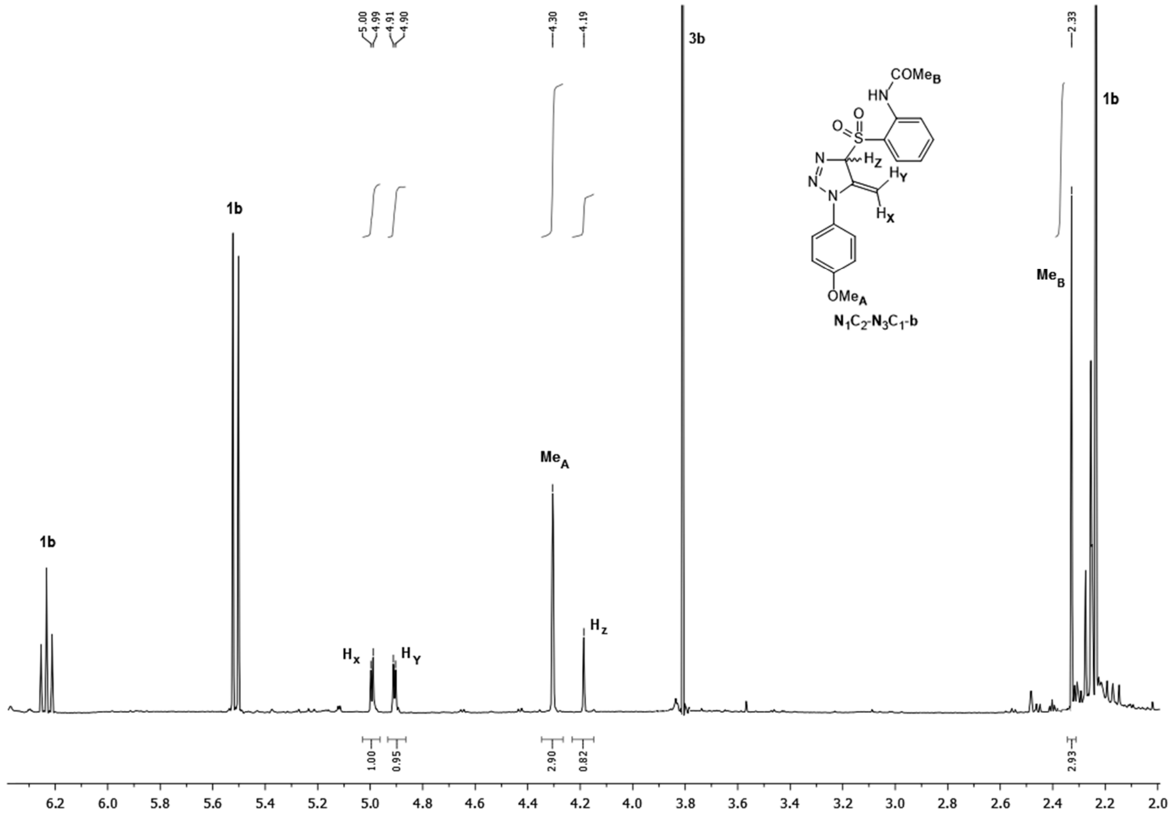

Global electron density transfer (M08-HX/pcseg-3//M08-HX/pcseg-2)

The global electron density transfer (GEDT) was calculated at the M08-HX/pcseg-3//M08-HX/pcseg-2 level using natural population analysis (NPA).

Table S1. Global electron density transfer (GEDT, in electrons) at the transition state of the investigated cycloadditions, calculated at the M08-HX/pcseg-3//M08-HX/pcseg-2 level. Positive values indicate that the azide transferred electron density to the allene.

	Isomer			
Reaction	N_1C_2-N_3C_1	N_1C_2-N_3C_1	N_1C_2-N_3C_3	N_1C_3-N_3C_2
**2a+3a**	–0.072	–0.093	–	–
**2b+3a**	+0.064	+0.087	–	–
**1a+3a**	+0.030	+0.081	$+2\cdot10^{-5}$	–0.005
**1b+3b**	+0.086	+0.186	+0.047	+0.033
**1b+3c**	+0.021	+0.138	+0.004	–0.009

The direction of the GEDT agrees with the analysis of the μ, ω, and *N* indices, except for the **1b+3c** reaction, which GEDT assigns to normal electron demand, whereas Δμ and Δω predict it has inverse electron demand. The GEDT from **3c** to **1b** is 0.48 to 0.65 electron lower than that from **3b**, even inducing an electron demand reversal in the N_1C_3-N_3C_2 TS. There is no correlation between the GEDT and the site- and regio-selectivity. In the **1b+3b** and **1a+3a** cases, the primary regioisomer comes from the TS with largest GEDT. Conversely, in the other cases, the primary regioisomer comes from the TS with the smallest (in absolute value) GEDT. Therefore, the energetic contribution related to the GEDT is not predominant in determining the reaction barriers and the selectivity.

Structure of the transition states (M08-HX/pcseg-2)

Relevant bond lengths are given in angstrom.

5a-N_1C_1-N_3C_2 TS

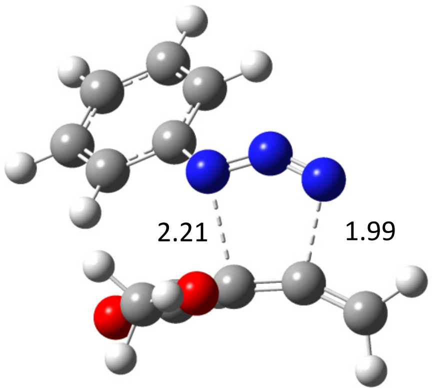

4a-N_1C_2-N_3C_1 TS

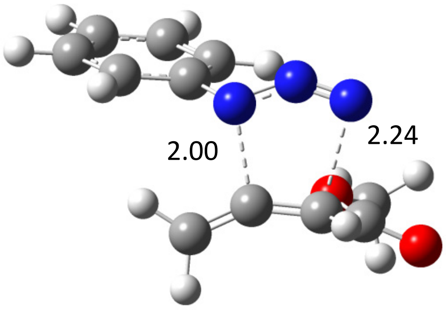

(1a+3a) N1C2-N3C3 TS

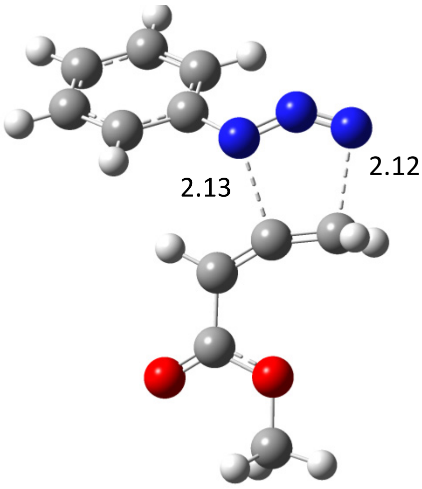

(1a+3a) N1C3-N3C2 TS

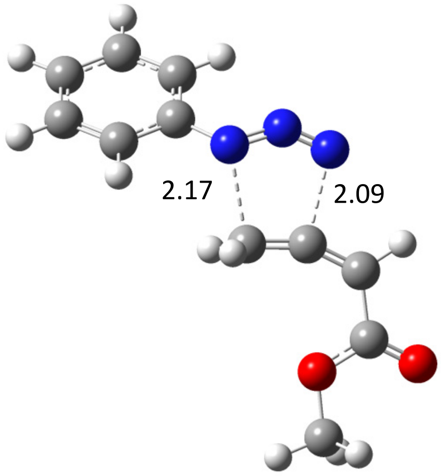

Figure S1. Structure of the transition states leading to the primary cycloadducts of the **1a** + **3a** cycloaddition calculated at the M08-HX/pcseg-2 level. Colour code: carbon, grey; nitrogen, blue; oxygen, red; hydrogen, light grey).

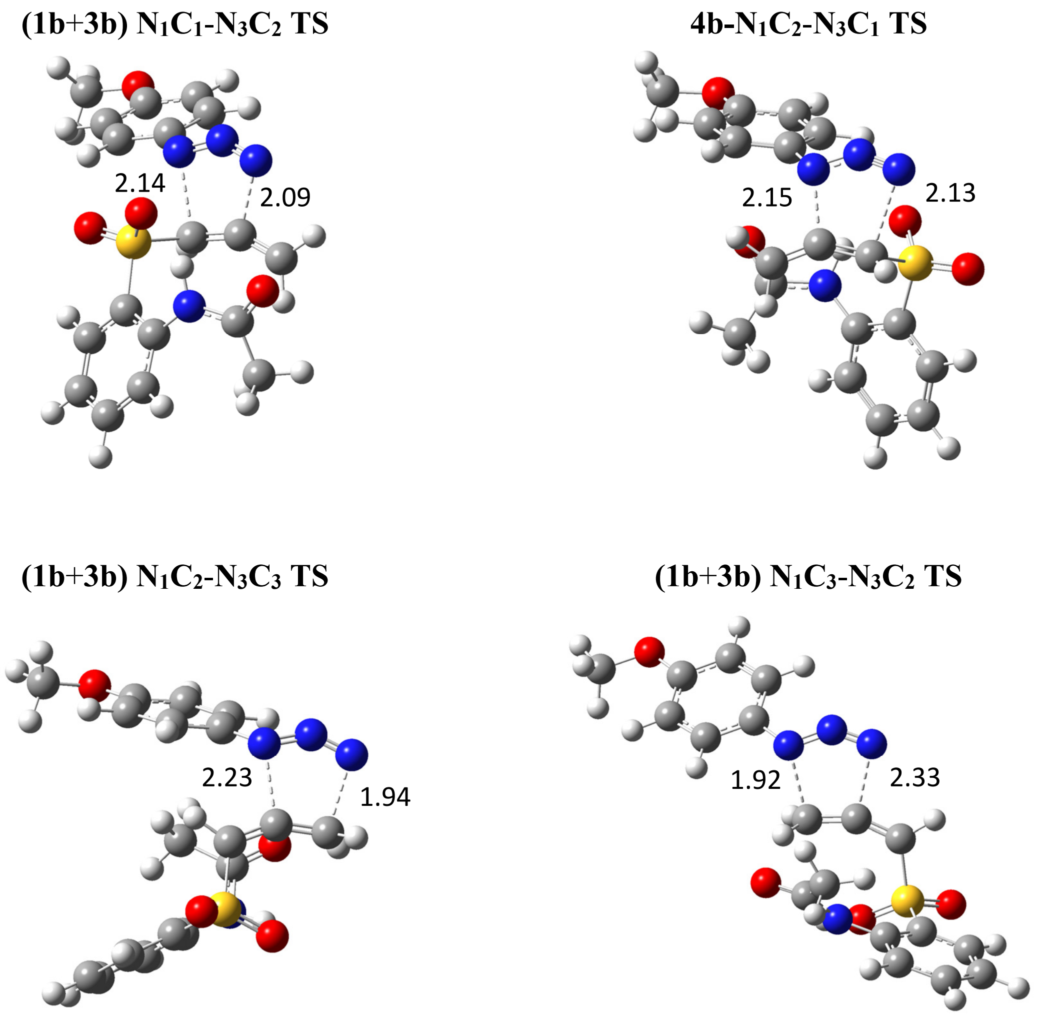

Figure S2. Structure of the transition states leading to the primary cycloadducts of the **1b** + **3b** cycloaddition calculated at the M08-HX/pcseg-2 level. Colour code: carbon, grey; nitrogen, blue; oxygen, red; sulphur, yellow; hydrogen, light grey).

(1b+3c) N_1C_1-N_3C_2 TS

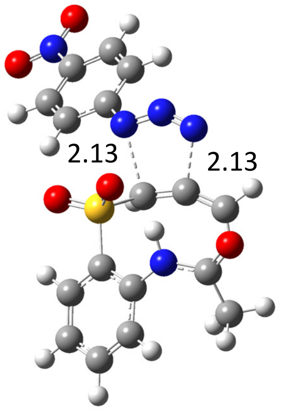

(1b+3c) N_1C_2-N_3C_1 TS

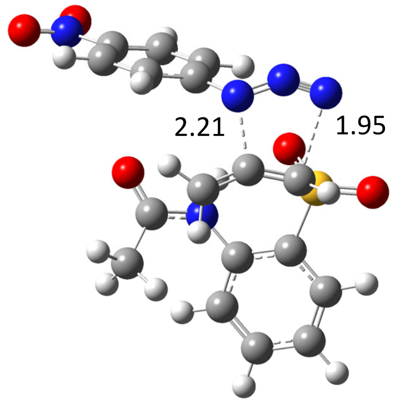

6-N_1C_2-N_3C_3 TS

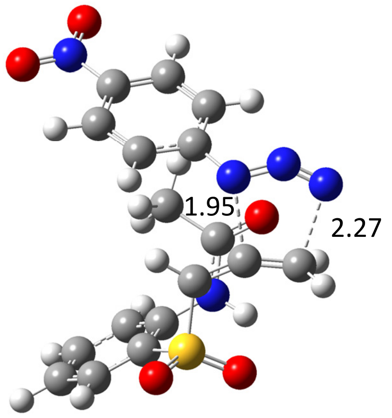

(1b+3c) N_1C_3-N_3C_2 TS

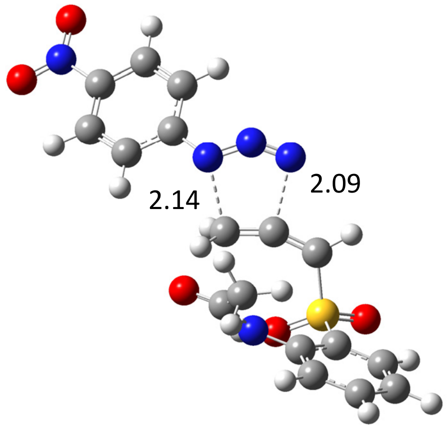

Figure S3. Structure of the transition states leading to the primary cycloadducts of the **1b** + **3c** cycloaddition calculated at the M08-HX/pcseg-2 level. Colour code: carbon, grey; nitrogen, blue; oxygen, red; sulphur, yellow; hydrogen, light grey).

6-N_1C_1-N_3C_2 TS

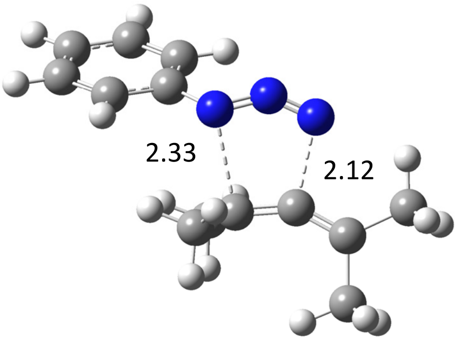

(2a+3a) N_1C_2-N_3C_1 TS

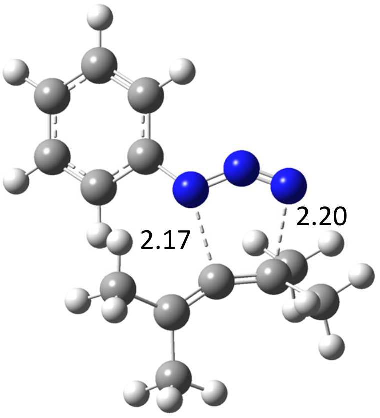

Figure S4. Structure of the transition states leading to the primary cycloadducts of the **2a** + **3a** cycloaddition calculated at the M08-HX/pcseg-2 level. Colour code: carbon, grey; nitrogen, blue; hydrogen, light grey).

7-N_1C_1-N_3C_2 TS

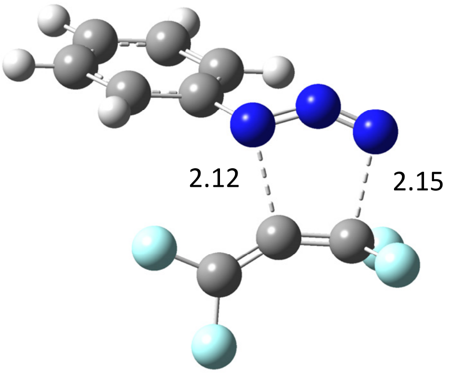

8-N_1C_2-N_3C_1 TS

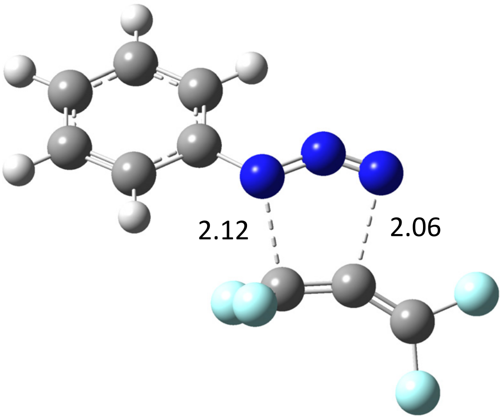

Figure S5. Structure of the transition states leading to the primary cycloadducts of the **2b** + **3a** cycloaddition calculated at the M08-HX/pcseg-2 level. Colour code: carbon, grey; nitrogen, blue; fluorine, light blue; hydrogen, light grey).

Cartesian coordinates of the transition states (M08-HX/pcseg-2)

All cartesian coordinates are given in angstrom.

```
1a+3a N1C1_N3C2 M08HX/pcseg-2
-----------------------------------------------------
      N    -0.942846     1.743318    -3.151618
      N    -0.584963     2.751959    -2.505225
      N    -0.616888     3.152257    -1.426783
      C    -1.491536     0.609284    -1.337506
      C    -1.246061     1.565743    -0.399650
      C    -1.199341     1.979101     0.840816
      H    -0.773048    -0.179993    -1.537465
      H    -0.869536     2.982156     1.089304
      H    -1.503893     1.321339     1.649752
      C    -0.044063     1.139796    -4.054157
      C    -0.462214    -0.059517    -4.627035
      C     1.206478     1.673905    -4.357164
      C     0.384907    -0.726372    -5.496925
      C     2.034797     1.001865    -5.243027
      C     1.631662    -0.198734    -5.812764
      H    -1.438364    -0.457899    -4.365351
      H     1.516294     2.614561    -3.912845
      H     0.064305    -1.665164    -5.936844
      H     3.005130     1.421979    -5.487694
      H     2.285998    -0.720350    -6.502902
      C    -2.842411     0.365112    -1.892575
      O    -3.143527    -0.612838    -2.525750
      O    -3.691376     1.359006    -1.624378
      C    -4.995953     1.198466    -2.167962
      H    -5.465760     0.292249    -1.778603
      H    -5.560151     2.079484    -1.866744
      H    -4.949765     1.128911    -3.257451
```

1a+3a N1C2_N3C1 M08HX/pcseg-2

N	-0.765681	3.869896	-1.751471
N	-0.771965	3.093437	-2.744101
N	-0.982840	2.006880	-3.015403
C	-1.934957	1.537897	-1.044134
C	-1.679486	2.712987	-0.397092
C	-1.740136	3.442022	0.686480
H	-1.348222	0.650955	-0.837982
H	-1.315633	4.437500	0.749894
H	-2.249259	3.051251	1.562048
C	-1.250941	5.190853	-1.927088
C	-0.716773	6.184375	-1.116112
C	-2.267275	5.485567	-2.832305
C	-1.207757	7.479658	-1.205983
C	-2.736459	6.784821	-2.928490
C	-2.213380	7.783078	-2.112916
H	0.091626	5.932877	-0.436999
H	-2.685943	4.690830	-3.443342
H	-0.791695	8.256970	-0.573466
H	-3.522756	7.020416	-3.638409
H	-2.589407	8.797895	-2.188544
C	-3.209621	1.313580	-1.742727
O	-3.674353	0.240922	-2.016334
O	-3.809395	2.476568	-2.071812
C	-5.055282	2.334692	-2.740661
H	-5.418172	3.344890	-2.926895
H	-4.928697	1.793678	-3.681783
H	-5.766832	1.786663	-2.118409

1a+3a N1C2_N3C3 M08HX/pcseg-2

N	0.686211	2.548876	0.189393
N	0.602105	3.374941	1.129464
N	-0.159891	4.108009	1.575620
C	-1.524960	1.417223	-1.017375
C	-1.395400	2.489446	-0.272651
C	-1.838641	3.577009	0.385179
H	-0.720078	0.709366	-1.183836
H	-2.488220	3.451077	1.243618
H	-1.859106	4.541902	-0.111528
C	1.451024	1.378717	0.343990
C	1.789580	0.689326	-0.817846
C	1.831961	0.881093	1.590119
C	2.488266	-0.505615	-0.730286
C	2.546703	-0.302762	1.663127
C	2.870385	-1.004812	0.507337
H	1.509107	1.109001	-1.778602
H	1.573351	1.428778	2.491248
H	2.745507	-1.042389	-1.637565
H	3.424712	-1.934789	0.573085
H	2.850080	-0.683877	2.632995
C	-2.795882	1.059484	-1.696702
O	-2.918127	0.080148	-2.382589
O	-3.787473	1.926626	-1.473345
C	-5.015154	1.606052	-2.115737
H	-5.383234	0.634100	-1.778339
H	-5.715516	2.393791	-1.842757
H	-4.883973	1.570921	-3.199963

1a+3a N1C3_N3C2 M08HX/pcseg-2

N	-0.369162	4.310973	1.171315
N	0.417435	3.445284	0.720788
N	0.387714	2.460607	0.135096
C	-1.698533	1.331482	-1.120161
C	-1.625907	2.440255	-0.419698
C	-2.120058	3.566603	0.126167
H	-0.844842	0.676081	-1.255037
H	-2.702717	3.518433	1.039737
H	-2.212884	4.472370	-0.467152
C	-0.040850	5.673463	1.054909
C	-0.986001	6.580437	1.529826
C	1.141179	6.132406	0.476236
C	-0.751474	7.940770	1.414750
C	1.369350	7.496572	0.380750
C	0.426903	8.405739	0.843489
H	-1.895682	6.202387	1.985250
H	1.878379	5.421376	0.116812
H	-1.492352	8.643575	1.781968
H	2.294355	7.850974	-0.062806
H	0.611535	9.471373	0.762756
C	-2.941936	0.873730	-1.789919
O	-3.008722	-0.139304	-2.432842
O	-3.981104	1.696583	-1.610731
C	-5.183962	1.281655	-2.245301
H	-5.500457	0.305684	-1.869334
H	-5.929434	2.039725	-2.010125
H	-5.042838	1.207936	-3.326378

1b+3b N1C1_N3C2 M08HX/pcseg-2

N	-1.210367	1.684746	-3.295436
N	-0.957748	2.752478	-2.705203
N	-1.031569	3.220926	-1.654308
C	-1.688741	0.657723	-1.378948
C	-1.477143	1.701629	-0.532047
C	-1.409166	2.187518	0.683196
H	-0.976939	-0.145826	-1.539252
H	-1.146412	3.223705	0.865760
H	-1.636102	1.556715	1.539379
C	-0.220295	1.098078	-4.114741
C	-0.613202	0.042671	-4.924849
C	1.113417	1.514612	-4.108689
C	0.317139	-0.609476	-5.722246
C	2.035208	0.880214	-4.912648
C	1.645574	-0.190419	-5.722590
O	2.618649	-0.753055	-6.471402
C	2.256567	-1.822302	-7.313001
S	-3.309181	0.302774	-1.970204
O	-4.037010	1.544064	-2.070394
O	-3.184617	-0.560167	-3.113848
C	-4.069956	-0.676231	-0.688217
C	-4.140381	-2.045357	-0.910187
C	-4.611822	-0.100261	0.474364
C	-4.728526	-2.878693	0.026636
C	-5.239398	-0.954004	1.384437
C	-5.279168	-2.322019	1.172520
N	-4.572254	1.277170	0.679997
C	-4.351569	1.978872	1.843501
O	-4.360103	3.187038	1.824712
C	-4.033489	1.219240	3.112983
H	-1.652190	-0.268852	-4.907429
H	1.420934	2.348236	-3.484148
H	-0.008975	-1.434712	-6.342998
H	3.072483	1.195494	-4.934455
H	3.164209	-2.130552	-7.830880
H	1.864749	-2.668879	-6.736090
H	1.506966	-1.512118	-8.051103
H	-3.731637	-2.438199	-1.835143
H	-4.776800	-3.947453	-0.147953
H	-5.733368	-0.528636	2.249410
H	-5.774044	-2.955574	1.901896
H	-4.561960	1.857240	-0.152227
H	-4.949230	1.024743	3.677146
H	-3.532974	0.266896	2.928590
H	-3.403377	1.869254	3.719887

1b+3b N1C2_N3C1 M08HX/pcseg-2

N	-0.413604	3.554860	-2.033558
N	-0.355367	3.073862	-3.197533
N	-0.355792	2.100532	-3.778160
C	-0.976878	0.948923	-1.851827
C	-0.780998	1.990420	-0.990033
C	-0.709678	2.371576	0.261933
H	-0.282202	0.127985	-1.972637
H	-0.445316	3.381123	0.555444
H	-0.956115	1.658355	1.044039
C	-1.206718	4.718037	-1.810611
C	-0.803257	5.594284	-0.819107
C	-2.385947	4.955165	-2.519369
C	-1.584332	6.694999	-0.492917
C	-3.150986	6.058092	-2.215613
C	-2.767312	6.924002	-1.187242
O	-3.598549	7.958904	-0.943726
C	-3.274113	8.815164	0.124868
S	-2.565353	0.667946	-2.507330
O	-3.144039	1.948778	-2.864704
O	-2.467217	-0.373103	-3.490642
C	-3.565082	0.028325	-1.170418
C	-3.785980	-1.342531	-1.169181
C	-4.141756	0.854120	-0.189589
C	-4.563047	-1.929139	-0.183994
C	-4.956645	0.247714	0.768233
C	-5.148403	-1.124060	0.782868
N	-3.957270	2.240127	-0.197630
C	-3.664019	3.071615	0.855085
O	-3.467047	4.250185	0.658499
C	-3.546579	2.486708	2.245375
H	0.136668	5.419376	-0.305101
H	-2.700306	4.262168	-3.293526
H	-1.259758	7.360124	0.297056
H	-4.072510	6.264512	-2.748355
H	-4.075250	9.551027	0.187649
H	-3.213815	8.262324	1.070026
H	-2.323449	9.333389	-0.052462
H	-3.342580	-1.934128	-1.963574
H	-4.728074	-3.000629	-0.186105
H	-5.471413	0.873114	1.488133
H	-5.787077	-1.561772	1.543577
H	-3.808887	2.668009	-1.105427
H	-4.512918	2.532410	2.754492
H	-3.206457	1.449934	2.247880
H	-2.848982	3.117030	2.796435

1b+3b N1C2_N3C3 M08HX/pcseg-2

N	0.378454	2.701501	-0.034892
N	0.349880	3.716182	0.705794
N	-0.371666	4.539248	1.044116
C	-1.874363	1.634741	-1.199768
C	-1.705394	2.662561	-0.409109
C	-2.162238	3.727160	0.276774
H	-1.097529	0.997632	-1.608618
H	-2.548545	3.578783	1.280887
H	-2.465859	4.616584	-0.264558
C	1.020303	1.537842	0.437559
C	1.358547	0.568415	-0.495270
C	1.295304	1.317038	1.790703
C	1.940271	-0.630011	-0.095338
C	1.907065	0.148595	2.187162
C	2.223025	-0.841035	1.250958
O	2.803142	-1.959630	1.739922
C	3.153238	-2.965563	0.819706
S	-3.512893	1.264752	-1.761232
O	-4.436520	2.155458	-1.105867
O	-3.487100	1.167280	-3.195243
C	-3.824068	-0.372168	-1.135419
C	-4.106865	-1.381639	-2.042984
C	-3.832087	-0.607333	0.242045
C	-4.405028	-2.654275	-1.579403
C	-4.157282	-1.882620	0.691233
C	-4.430426	-2.898948	-0.212694
N	-3.537255	0.430694	1.153328
C	-2.383583	0.517325	1.896369
O	-2.108666	1.538874	2.488592
C	-1.466230	-0.679754	1.894412
H	1.185483	0.765675	-1.548544
H	1.018955	2.065531	2.527211
H	2.190736	-1.371694	-0.843643
H	2.140673	-0.034183	3.230630
H	3.602444	-3.772682	1.397366
H	2.271053	-3.347784	0.291314
H	3.881115	-2.598754	0.085695
H	-4.089835	-1.147435	-3.102697
H	-4.628463	-3.449020	-2.282703
H	-4.212601	-2.060438	1.760375
H	-4.682732	-3.887645	0.157083
H	-3.989473	1.321564	0.982017
H	-1.991586	-1.624064	2.028557
H	-0.943778	-0.718833	0.929195
H	-0.730077	-0.543587	2.682790

1b+3b N1C3_N3C2 M08HX/pcseg-2

N	-0.089256	4.016041	1.598494
N	0.664202	3.236349	0.978913
N	0.616535	2.353669	0.249492
C	-1.630434	1.475852	-1.015568
C	-1.465946	2.453131	-0.162756
C	-1.885475	3.500843	0.570550
H	-0.857144	0.795183	-1.356559
H	-2.435717	3.340110	1.495706
H	-1.978292	4.477008	0.101501
C	0.240124	5.381400	1.717402
C	-0.588347	6.156758	2.514866
C	1.311998	5.976997	1.049345
C	-0.367441	7.521490	2.644803
C	1.546276	7.328308	1.191701
C	0.706590	8.113303	1.985580
O	1.012118	9.428381	2.057798
C	0.185196	10.244133	2.852715
S	-3.214395	1.224915	-1.763119
O	-4.093240	2.297223	-1.361844
O	-3.010711	0.937183	-3.156111
C	-3.803903	-0.267619	-0.995836
C	-3.888740	-1.406821	-1.782340
C	-4.176211	-0.284674	0.356856
C	-4.340561	-2.596141	-1.232780
C	-4.656427	-1.482622	0.884509
C	-4.722575	-2.624568	0.101416
N	-4.104698	0.884475	1.119927
C	-3.682254	1.052668	2.414410
O	-3.693225	2.160544	2.908634
C	-3.169183	-0.142169	3.182178
H	-1.414471	5.681493	3.034362
H	1.967328	5.373924	0.428158
H	-1.032984	8.106071	3.267722
H	2.379743	7.807266	0.689706
H	0.586984	11.254506	2.784027
H	-0.848770	10.241592	2.486369
H	0.196187	9.921999	3.901230
H	-3.596198	-1.335583	-2.825113
H	-4.406795	-3.488754	-1.844481
H	-5.010372	-1.506981	1.908202
H	-5.100628	-3.543410	0.538395
H	-4.284046	1.751897	0.624095
H	-3.988607	-0.611784	3.732597
H	-2.706489	-0.892722	2.540114
H	-2.449170	0.230695	3.909553

1b+3c N1C1_N3C2 M08HX/pcseg-2

N	-1.123760	1.612260	-3.226846
N	-0.874735	2.684182	-2.628836
N	-0.974622	3.127039	-1.571887
C	-1.667448	0.567962	-1.356630
C	-1.442615	1.583154	-0.480989
C	-1.371105	2.046788	0.742214
H	-0.982868	-0.259691	-1.516723
H	-1.091779	3.074777	0.945191
H	-1.614925	1.404306	1.585014
C	-0.152911	1.074421	-4.088953
C	-0.454881	-0.152076	-4.682568
C	1.063504	1.712000	-4.344635
C	0.470181	-0.750151	-5.517246
C	1.981736	1.118629	-5.190300
C	1.672081	-0.105407	-5.757400
N	2.653475	-0.739320	-6.649488
O	3.689374	-0.147546	-6.848362
O	2.362323	-1.812199	-7.124904
S	-3.306075	0.280154	-1.964497
O	-3.968793	1.555377	-2.069864
O	-3.201380	-0.582780	-3.109505
C	-4.109127	-0.666941	-0.688866
C	-4.221847	-2.033823	-0.912095
C	-4.647220	-0.070477	0.465870
C	-4.849815	-2.845641	0.016880
C	-5.315125	-0.903020	1.366759
C	-5.397359	-2.268952	1.154550
N	-4.564253	1.304415	0.672787
C	-4.321265	1.999005	1.837611
O	-4.261816	3.205274	1.809854
C	-4.067750	1.231354	3.115930
H	-1.414955	-0.611906	-4.474571
H	1.279509	2.674474	-3.893087
H	0.270685	-1.703079	-5.992581
H	2.932364	1.587292	-5.415279
H	-3.816300	-2.441962	-1.831696
H	-4.931776	-3.912185	-0.158096
H	-5.807339	-0.461156	2.224297
H	-5.923833	-2.884731	1.876942
H	-4.519603	1.884715	-0.157644
H	-5.005539	1.074161	3.654984
H	-3.601247	0.259080	2.946289
H	-3.428857	1.856772	3.739087

1b+3c N1C2_N3C1 M08HX/pcseg-2

N	-0.402734	3.560000	-2.041328
N	-0.376486	3.079084	-3.209739
N	-0.393582	2.090527	-3.766653
C	-0.984209	0.953840	-1.888650
C	-0.790113	1.965658	-0.994678
C	-0.734294	2.329499	0.262019
H	-0.291739	0.132968	-2.025963
H	-0.472556	3.332415	0.578654
H	-0.986615	1.602169	1.029550
C	-1.201119	4.712542	-1.806522
C	-0.809418	5.550200	-0.768558
C	-2.355771	4.969953	-2.543537
C	-1.594398	6.638915	-0.432878
C	-3.131924	6.067348	-2.222180
C	-2.741350	6.872140	-1.166095
N	-3.581048	8.028464	-0.810932
O	-3.214396	8.720772	0.109847
O	-4.580788	8.212133	-1.464858
S	-2.590337	0.676922	-2.525364
O	-3.159415	1.964034	-2.874774
O	-2.500791	-0.358693	-3.513325
C	-3.563739	0.037590	-1.172385
C	-3.758409	-1.337679	-1.156079
C	-4.143535	0.862986	-0.192354
C	-4.511116	-1.928210	-0.155008
C	-4.933004	0.250745	0.782628
C	-5.097976	-1.124234	0.812110
N	-3.986865	2.251610	-0.223203
C	-3.690711	3.109957	0.806462
O	-3.484686	4.280334	0.570184
C	-3.586618	2.569954	2.213923
H	0.118359	5.350589	-0.243639
H	-2.649897	4.298662	-3.343672
H	-1.329930	7.308419	0.376153
H	-4.040685	6.299015	-2.764247
H	-3.315784	-1.929598	-1.950657
H	-4.656898	-3.002369	-0.144738
H	-5.451273	0.872563	1.502887
H	-5.718376	-1.566378	1.585213
H	-3.847693	2.662895	-1.139496
H	-4.562875	2.613440	2.704110
H	-3.230585	1.539053	2.251348
H	-2.910034	3.227108	2.759483

1b+3c N1C2_N3C3 M08HX/pcseg-2

N	0.385658	2.692148	0.040598
N	0.309420	3.682674	0.818830
N	-0.441628	4.491453	1.121060
C	-1.862200	1.622020	-1.222618
C	-1.698277	2.657510	-0.443324
C	-2.165976	3.728581	0.226435
H	-1.093311	0.962077	-1.607150
H	-2.608434	3.579302	1.207016
H	-2.430701	4.623537	-0.326637
C	1.026654	1.534628	0.496279
C	1.309308	0.555083	-0.458622
C	1.361994	1.328592	1.838129
C	1.887433	-0.640842	-0.073836
C	1.967795	0.147660	2.219448
C	2.204778	-0.824772	1.260933
N	2.827380	-2.090339	1.673195
O	3.013104	-2.924307	0.817219
O	3.109253	-2.217382	2.842214
S	-3.506181	1.262859	-1.789596
O	-4.417512	2.167677	-1.137358
O	-3.477198	1.159733	-3.221840
C	-3.822300	-0.367058	-1.150797
C	-4.112495	-1.380682	-2.051266
C	-3.826000	-0.593912	0.228585
C	-4.413818	-2.649059	-1.577718
C	-4.154196	-1.864068	0.687393
C	-4.434965	-2.884927	-0.209707
N	-3.516836	0.449532	1.131143
C	-2.352821	0.534693	1.854648
O	-2.048673	1.566115	2.416706
C	-1.461447	-0.682015	1.874477
H	1.100959	0.758146	-1.503353
H	1.133249	2.090733	2.575455
H	2.116546	-1.420447	-0.790548
H	2.243955	-0.040843	3.250379
H	-4.098359	-1.154556	-3.112751
H	-4.641676	-3.447731	-2.275010
H	-4.205956	-2.036024	1.757570
H	-4.688052	-3.870682	0.167060
H	-3.970475	1.339786	0.961594
H	-1.999364	-1.598891	2.112229
H	-1.008348	-0.810344	0.883205
H	-0.676156	-0.513013	2.607221

1b+3c N1C3_N3C2 M08HX/pcseg-2

N	-0.122992	4.089395	1.451670
N	0.628399	3.311445	0.818753
N	0.557691	2.421130	0.104570
C	-1.705117	1.481762	-1.073512
C	-1.538720	2.476588	-0.242673
C	-1.962817	3.526821	0.483270
H	-0.927684	0.812461	-1.426733
H	-2.473921	3.367238	1.431661
H	-2.087864	4.493360	0.001260
C	0.225959	5.428611	1.637694
C	-0.728420	6.230009	2.268542
C	1.442834	5.971572	1.218609
C	-0.473661	7.572060	2.470383
C	1.701845	7.312560	1.430767
C	0.739559	8.091647	2.049580
N	1.017185	9.516685	2.271126
O	0.158920	10.173369	2.813685
O	2.086164	9.940870	1.896476
S	-3.309164	1.196215	-1.771094
O	-4.187114	2.260443	-1.348615
O	-3.138096	0.904529	-3.166568
C	-3.848703	-0.298750	-0.975377
C	-3.943209	-1.443746	-1.752642
C	-4.176172	-0.312060	0.388932
C	-4.360280	-2.635125	-1.180833
C	-4.621002	-1.512700	0.939705
C	-4.697193	-2.660305	0.165605
N	-4.094355	0.864090	1.141789
C	-3.605595	1.051614	2.409638
O	-3.588450	2.168459	2.884519
C	-3.062509	-0.132583	3.171823
H	-1.660980	5.783047	2.596483
H	2.182288	5.339679	0.738495
H	-1.194153	8.220831	2.953990
H	2.636789	7.765068	1.122096
H	-3.686906	-1.375700	-2.805112
H	-4.434961	-3.532400	-1.784632
H	-4.939815	-1.535243	1.974937
H	-5.047603	-3.581542	0.620104
H	-4.309708	1.724000	0.647697
H	-3.861994	-0.599129	3.753361
H	-2.620292	-0.888687	2.521767
H	-2.319652	0.248669	3.871192

2a+3a N1C1_N3C2 M08HX/pcseg-2

N	-0.760088	1.791238	-3.172071
N	-0.226829	2.739688	-2.543987
N	-0.162109	3.158376	-1.477721
C	-1.203504	0.612435	-1.221864
C	-1.092682	1.663469	-0.368690
C	-1.273207	2.122125	0.853573
C	-0.027524	1.162314	-4.185315
C	-0.716635	0.250484	-4.985386
C	1.342828	1.352909	-4.376042
C	-0.037906	-0.472754	-5.953107
C	2.006875	0.635468	-5.358078
C	1.325062	-0.284032	-6.146155
H	-1.785359	0.131367	-4.840670
H	1.877077	2.069298	-3.759000
H	-0.581234	-1.181657	-6.569774
H	3.070345	0.794498	-5.506706
H	1.852107	-0.844459	-6.910642
C	-0.044783	-0.335274	-1.399078
H	-0.093919	-1.119701	-0.632982
H	-0.069324	-0.823049	-2.378659
H	0.913081	0.179694	-1.288228
C	-2.555293	0.119065	-1.668474
H	-2.497991	-0.359686	-2.650158
H	-2.920231	-0.632142	-0.955254
H	-3.281742	0.931013	-1.711480
C	-1.735229	1.173143	1.936576
H	-1.883685	0.161413	1.553473
H	-1.001216	1.134967	2.749675
H	-2.676989	1.523417	2.374101
C	-1.069721	3.535326	1.322767
H	-2.006569	3.923301	1.738583
H	-0.334609	3.557956	2.135284
H	-0.736218	4.200732	0.530189

2a+3a N1C2_N3C1 M08HX/pcseg-2

N	-1.257034	3.984532	-1.814164
N	-1.134665	3.100791	-2.696906
N	-1.206307	1.955979	-2.756682
C	-2.136569	1.558635	-0.852597
C	-1.703442	2.644011	-0.165865
C	-1.345019	3.231921	0.954248
C	-1.601319	5.298794	-2.167736
C	-2.216219	6.086027	-1.195515
C	-1.338585	5.835049	-3.429743
C	-2.556299	7.398422	-1.484407
C	-1.687642	7.147546	-3.704152
C	-2.292889	7.939081	-2.735995
H	-2.443437	5.646089	-0.230645
H	-0.858395	5.219288	-4.183101
H	-3.040206	8.001697	-0.722730
H	-1.479675	7.556282	-4.688125
H	-2.560705	8.966314	-2.957407
C	-0.458605	4.436030	1.094178
H	-0.980610	5.248029	1.613850
H	0.407305	4.175905	1.713503
H	-0.101113	4.801245	0.132504
C	-1.817206	2.638254	2.263307
H	-2.419676	3.366723	2.817808
H	-2.416337	1.739330	2.107676
H	-0.958699	2.384277	2.894767
C	-1.476387	0.217180	-0.628726
H	-2.021233	-0.342547	0.141597
H	-1.487959	-0.378797	-1.546124
H	-0.442158	0.332968	-0.300425
C	-3.532296	1.529727	-1.429022
H	-3.570599	0.895419	-2.319623
H	-4.237206	1.122792	-0.694351
H	-3.868588	2.533857	-1.700172

2b+3a N1C1_N3C2 M08HX/pcseg-2

N	-0.762679	1.720651	-3.017660
N	-0.418049	2.789960	-2.466704
N	-0.476118	3.329110	-1.463885
C	-1.403126	0.640726	-1.307112
C	-1.270690	1.631075	-0.408306
C	-1.396305	1.985850	0.832790
C	0.048198	1.128369	-4.003980
C	-0.430930	-0.048661	-4.572267
C	1.278865	1.651294	-4.393740
C	0.331262	-0.706988	-5.523078
C	2.023123	0.990273	-5.358298
C	1.557554	-0.190355	-5.922654
H	-1.395192	-0.433575	-4.255766
H	1.641959	2.573728	-3.951109
H	-0.040343	-1.627775	-5.960563
H	2.977961	1.402272	-5.668463
H	2.147320	-0.704158	-6.673939
F	-0.531940	-0.351273	-1.429605
F	-1.123789	3.171774	1.328097
F	-1.826087	1.210081	1.812290
F	-2.566252	0.270236	-1.819936

2b+3a N1C2_N3C1 M08HX/pcseg-2

N	-0.672248	3.947145	-1.733479
N	-0.776036	3.122826	-2.669221
N	-1.091092	2.035766	-2.855877
C	-1.969458	1.535445	-1.065776
C	-1.660655	2.649229	-0.372480
C	-1.644356	3.208072	0.798991
C	-1.238329	5.239374	-1.925949
C	-0.731082	6.276297	-1.153077
C	-2.278147	5.465338	-2.824187
C	-1.276937	7.545169	-1.272103
C	-2.807797	6.739628	-2.944258
C	-2.312609	7.780321	-2.166955
H	0.085144	6.072634	-0.469113
H	-2.665033	4.648182	-3.426123
H	-0.884426	8.356418	-0.668003
H	-3.614025	6.920708	-3.647455
H	-2.732807	8.775901	-2.262765
F	-1.089493	4.361928	1.103473
F	-2.194166	2.704890	1.887460
F	-1.334535	0.379342	-0.896012
F	-3.185298	1.309427	-1.558996

Energetics of the transition states

Table S2. Energetics of the isomeric TSs calculated using the M08_HX functional and the Jensen pcseg basis sets. $\Delta H_{TS} = E_{elec}$(pcseg-3//pcseg-2) + $H_{correct}$(pcseg-2). $\delta\Delta H^{\ddagger} = \Delta H_{TS} - \min(\Delta H_{TS})$.

Reaction	Isomer		T (K)	ΔH_{TS} (hartree)	$\delta\Delta H^{\ddagger}$ (kJ/mol)	k relative	Regio-select.	Site-select.	E pc3//pc2 (hartree)	H correct/pc2 (hartree)
1a+3a	N1C1	N3C2	347	-740.229	2.0	0.492	27%		-740.458	0.228954
1a+3a	N1C2	N3C1	347	-740.230	0.0	1.000	54%	81%	-740.459	0.228747
1a+3a	N1C2	N3C3	347	-740.228	4.1	0.241	13%		-740.457	0.228738
1a+3a	N1C3	N3C2	347	-740.227	6.1	0.121	7%	19%	-740.456	0.228866
1b+3c	N1C1	N3C2	338	-1704.509	15.4	0.004	0%		-1704.859	0.349869
1b+3c	N1C2	N3C1	338	-1704.515	0.0	1.000	51%	51%	-1704.865	0.349666
1b+3c	N1C2	N3C3	338	-1704.514	1.6	0.556	28%		-1704.864	0.349378
1b+3c	N1C3	N3C2	338	-1704.514	2.6	0.398	20%	49%	-1704.864	0.349782
1b+3b	N1C1	N3C2	338	-1614.503	15.7	0.004	0%		-1614.882	0.379724
1b+3b	N1C2	N3C1	338	-1614.509	0.0	1.000	66%	66%	-1614.888	0.379462
1b+3b	N1C2	N3C3	338	-1614.508	2.8	0.373	25%		-1614.887	0.379174
1b+3b	N1C3	N3C2	338	-1614.506	5.6	0.138	9%	34%	-1614.886	0.379709
2a+3a	N1C1	N3C2	361	-669.554	0.0	1.000	96%		-669.855	0.301406
2a+3a	N1C2	N3C1	361	-669.550	9.5	0.042	4%		-669.852	0.301539
2b+3a	N1C1	N3C2	323	-909.381	0.0	1.000	100%		-909.532	0.151080
2b+3a	N1C2	N3C1	323	-909.372	24.9	0.000	0%		-909.523	0.151064

Table S3. Energetics of the isomeric TSs calculated using the ωB97X-D functional and the Jensen pcseg basis sets. $\Delta H_{TS} = E_{elec}$(pcseg-3//pcseg-2) + $H_{correct}$(pcseg-2). $\delta\Delta H^{‡} = \Delta H_{TS} - \min(\Delta H_{TS})$.

Reaction	Isomer		T (K)	ΔH_{TS} (hartree)	$\delta\Delta H^{‡}$ (kJ/mol)	*k* relative	Regio-select.	Site-select.	*E* pc3//pc2 (hartree)	*H* correct/pc2 (hartree)
1a+3a	N1C1	N3C2	347	-740.180	2.4	0.43	24%		-740.409	0.229500
1a+3a	N1C2	N3C1	347	-740.181	0.0	1.00	56%	81%	-740.410	0.229351
1a+3a	N1C2	N3C3	347	-740.179	4.1	0.24	13%		-740.409	0.229373
1a+3a	N1C3	N3C2	347	-740.178	6.5	0.11	6%	19%	-740.408	0.229435
1b+3c	N1C1	N3C2	338	-1704.467	19.6	0.00	0%		-1704.818	0.350982
1b+3c	N1C2	N3C1	338	-1704.473	5.4	0.15	8%	9%	-1704.823	0.350661
1b+3c	N1C2	N3C3	338	-1704.475	0.0	1.00	58%		-1704.825	0.350616
1b+3c	N1C3	N3C2	338	-1704.474	1.5	0.58	34%	91%	-1704.825	0.350742
1b+3b	N1C1	N3C2	338	-1614.446	19.5	0.00	0%		-1614.827	0.381180
1b+3b	N1C2	N3C1	338	-1614.452	3.9	0.25	17%	17%	-1614.832	0.380874
1b+3b	N1C2	N3C3	338	-1614.453	0.0	1.00	69%		-1614.834	0.380797
1b+3b	N1C3	N3C2	338	-1614.451	4.4	0.21	14%	83%	-1614.832	0.380983
2a+3a	N1C1	N3C2	361	-669.483	0.0	1.00	97%		-669.785	0.302752
2a+3a	N1C2	N3C1	361	-669.479	10.0	0.04	3%		-669.782	0.303008
2b+3a	N1C1	N3C2	323	-909.379	0.0	1.00	100%		-909.530	0.151341
2b+3a	N1C2	N3C1	323	-909.370	22.9	0.00	0%		-909.522	0.151289

Table S4. Energetics of the isomeric TSs calculated using the B3LYP functional and the Jensen pcseg basis sets. $\Delta H_{TS} = E_{elec}$(pcseg-3//pcseg-2) + $H_{correct}$(pcseg-2). $\delta\Delta H^{‡} = \Delta H_{TS} - \min(\Delta H_{TS})$.

Reaction	Isomer		T (K)	ΔH_{TS} (hartree)	$\delta\Delta H^{‡}$ (kJ/mol)	*k* relative	Regio-select.	Site-select.	*E* pc3//pc2 (hartree)	*H* correct/pc2 (hartree)
1a+3a	N1C1	N3C2	347	-740.459	1.6	0.571	24%		-740.686	0.227039
1a+3a	N1C2	N3C1	347	-740.458	3.6	0.289	12%	37%	-740.685	0.226935
1a+3a	N1C2	N3C3	347	-740.459	2.0	0.493	21%		-740.686	0.226963
1a+3a	N1C3	N3C2	347	-740.460	0.0	1.000	43%	63%	-740.687	0.226968
1b+3c	N1C1	N3C2	338	-1704.947	23.5	0.000	0%		-1705.293	0.346706
1b+3c	N1C2	N3C1	338	-1704.945	28.1	0.000	0%	0%	-1705.291	0.346570
1b+3c	N1C2	N3C3	338	-1704.952	8.8	0.044	4%		-1705.299	0.346667
1b+3c	N1C3	N3C2	338	-1704.955	0.0	1.000	96%	100%	-1705.302	0.346731
1b+3b	N1C1	N3C2	338	-1614.895	19.7	0.001	0%		-1615.271	0.376741
1b+3b	N1C2	N3C1	338	-1614.895	19.4	0.001	0%	0%	-1615.272	0.376677
1b+3b	N1C2	N3C3	338	-1614.900	6.0	0.118	11%		-1615.277	0.376690
1b+3b	N1C3	N3C2	338	-1614.902	0.0	1.000	89%	100%	-1615.279	0.376793
2a+3a	N1C1	N3C2	361	-669.735	0.0	1.000	99%		-670.035	0.299874
2a+3a	N1C2	N3C1	361	-669.730	13.7	0.010	1%		-670.030	0.300073
2b+3a	N1C1	N3C2	323	-909.687	0.0	1.000	100%		-909.837	0.149473
2b+3a	N1C2	N3C1	323	-909.679	22.3	0.000	0%		-909.828	0.149391

Table S5. Activation Gibbs free energy difference between isomeric TSs ($\delta\Delta G^{\ddagger}$) and corresponding isomeric ratio (*Y*), calculated using the M08-HX, ωB97X-D, and B3LYP functionals and compared to the experimental isomeric ratio.

		M08-HX		ωB97X-D		B3LYP		Exp
Reaction	Isomer	$\delta\Delta G^{\ddagger}$ (kJ/mol)	*Y* (%)	$\delta\Delta G^{\ddagger}$ (kJ/mol)	*Y* (%)	$\delta\Delta G^{\ddagger}$ (kJ/mol)	*Y* (%)	*Y* (%)
2a+3a	N_1C_1-N_3C_2	0.0	98	0.0	99	0.0	100	100
	N_1C_2-N_3C_1	12.3	2	15.5	1	24.7	0	0
2b+3a	N_1C_1-N_3C_2	0.0	100	0.0	100	0.0	100	89
	N_1C_2-N_3C_1	27.5	0	25.2	0	25.8	0	11
1a+3a	N_1C_1-N_3C_2	2.0	21	2.0	20	4.3	14	10
	N_1C_2-N_3C_1	0.0	42	0.0	40	6.6	6	90
	N_1C_2-N_3C_3	2.0	21	2.0	20	3.9	17	0
	N_1C_3-N_3C_2	3.0	15	2.1	19	0.0	63	0
1b+3c	N_1C_1-N_3C_2	17.2	0	22.5	0	25.1	0	0
	N_1C_2-N_3C_1	10.2	2	15.5	0	28.4	0	0
	N_1C_2-N_3C_3	3.0	25	4.5	17	11.2	2	100
	N_1C_3-N_3C_2	0.0	73	0.0	83	0.0	98	0
1b+3b	N_1C_1-N_3C_2	14.6	0	21.8	0	20.3	0	0
	N_1C_2-N_3C_1	5.7	10	8.7	3	18.2	0	100
	N_1C_2-N_3C_3	4.2	16	0.0	69	6.8	8	0
	N_1C_3-N_3C_2	0.0	74	2.6	28	0.0	92	0

Table S6. Activation enthalpy difference between isomeric TSs ($\delta\Delta G^{\ddagger}$) and corresponding isomeric ratio (Y), calculated using the M08-HX functional with the PCM solvation model using the solvent parameters for benzonitrile.

		M08-HX		Exp
Reaction	Isomer	$\delta\Delta G^{\ddagger}$ (kJ/mol)	Y (%)	Y (%)
2a+3a	N_1C_1-N_3C_2	0.0	97	100
	N_1C_2-N_3C_1	10.3	3	0
2b+3a	N_1C_1-N_3C_2	0.0	100	89
	N_1C_2-N_3C_1	21.5	0	11
1a+3a	N_1C_1-N_3C_2	7.8	5	10
	N_1C_2-N_3C_1	0.0	83	90
	N_1C_2-N_3C_3	7.4	6	0
	N_1C_3-N_3C_2	8.1	5	0
1b+3c	N_1C_1-N_3C_2	12.3	1	0
	N_1C_2-N_3C_1	2.5	23	0
	N_1C_2-N_3C_3	2.6	22	100
	N_1C_3-N_3C_2	0.0	55	0
1b+3b	N_1C_1-N_3C_2	13.2	1	0
	N_1C_2-N_3C_1	0.0	70	100
	N_1C_2-N_3C_3	3.7	19	0
	N_1C_3-N_3C_2	5.4	10	0

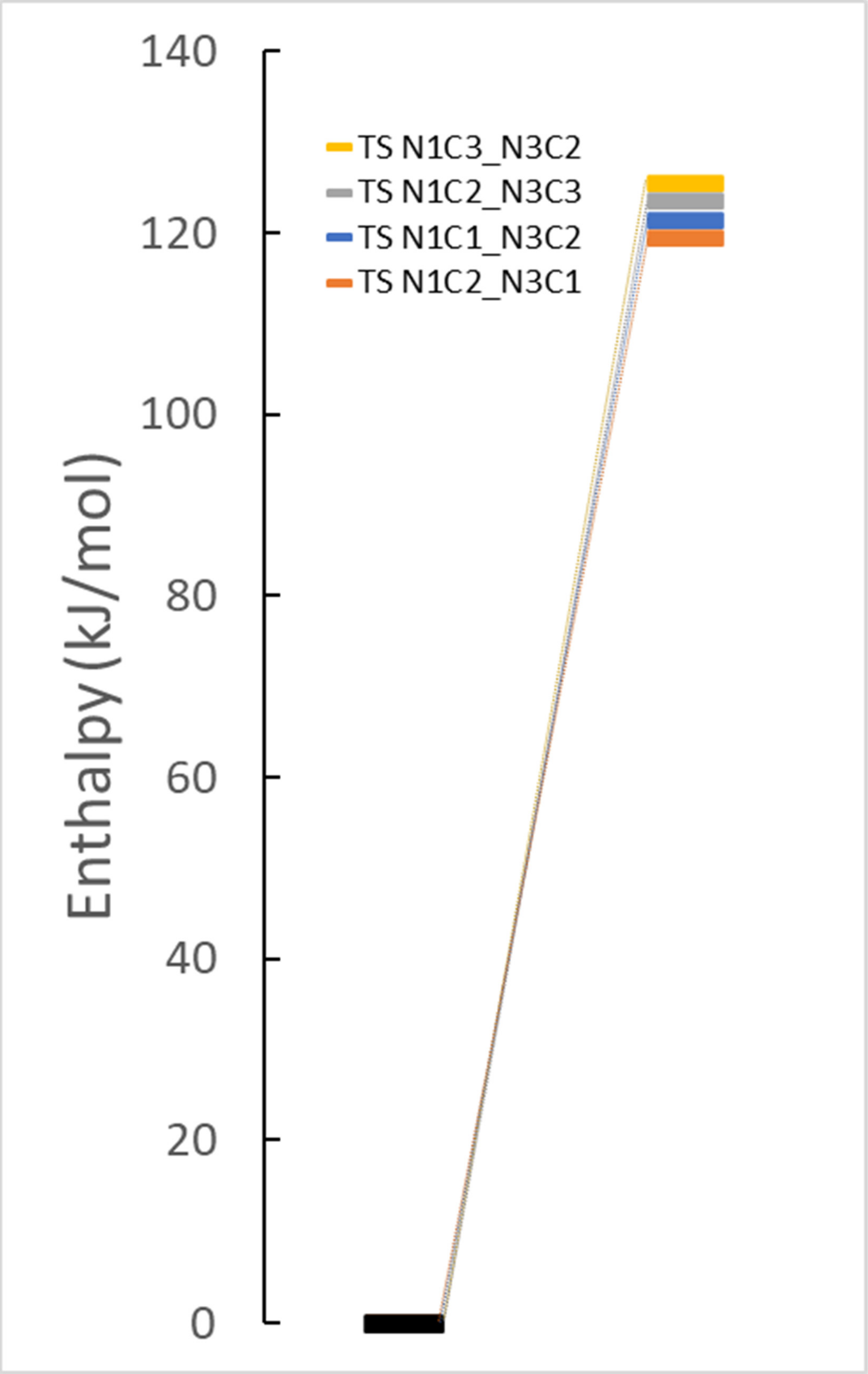

Figure S6. Schematic picture of the energetics of the **1a** + **3a**cycloaddition calculated at the M08-HX/pcseg-3//M08-HX/pcseg-2 level.
